# Reactivity of a Bis(alkynyl)-Vinylidene Osmium Complex: Access to Carbynes, Vinyl Ester Derivatives, and C–C and C–N Bond-Forming Reactions

**DOI:** 10.1021/acs.organomet.6c00277

**Published:** 2026-09-22

**Authors:** Sheila G. Curto, Joaquín M. Granados, Enrique Oñate, Montserrat Oliván

**Affiliations:** Departamento de Química Inorgánica - Instituto de Síntesis Química y Catálisis Homogénea (ISQCH) - Centro de Innovación en Química Avanzada (ORFEO−CINQA), Universidad de Zaragoza - CSIC, 50009 Zaragoza, Spain

## Abstract

Hexahydride OsH_6_(P^i^Pr_3_)_2_ (**1**) reacts with *tert*-butylacetylene to yield bis­(alkynyl)-vinylidene Os­(C≡C^t^Bu)_2_(=C=CH^t^Bu)­(P^i^Pr_3_)_2_ (**2**). Subsequent protonation of **2** with HOTf or Et_3_N·3HF affords the bis­(alkynyl)-carbyne derivatives [Os­(C≡C^t^Bu)_2_(≡CCH_2_^t^Bu)­(P^i^Pr_3_)_2_]­OTf (**3**) and OsF­(C≡C^t^Bu)_2_(≡CCH_2_^t^Bu)­(P^i^Pr_3_)_2_ (**4**), respectively. In contrast, treatment of **2** with acetic acid leads to the alkynyl-vinylidene-vinyl ester derivative Os­(C≡C^t^Bu)­{κ^2^-*O,C*-OC­(CH_3_)­OC=CH^t^Bu}­(=C=CH^t^Bu)­(P^i^Pr_3_)_2_ (**5**). Density functional theory (DFT) calculations, together with deuterium labeling experiments, demonstrate that the protonation may occur either at the C_β_ carbon of the vinylidene ligand or at the C_β_ carbon atom of one of the alkynyl groups, and that the formation of **5** is governed by thermodynamic control. The reaction of **2** with 2-aminopyridine provides a new route for the selective synthesis of a 2,3-disubstituted imidazo­[1,2-*a*]­pyridine derivative starting from *tert*-butylacetylene and 2-aminopyridine.

## Graphical abstract

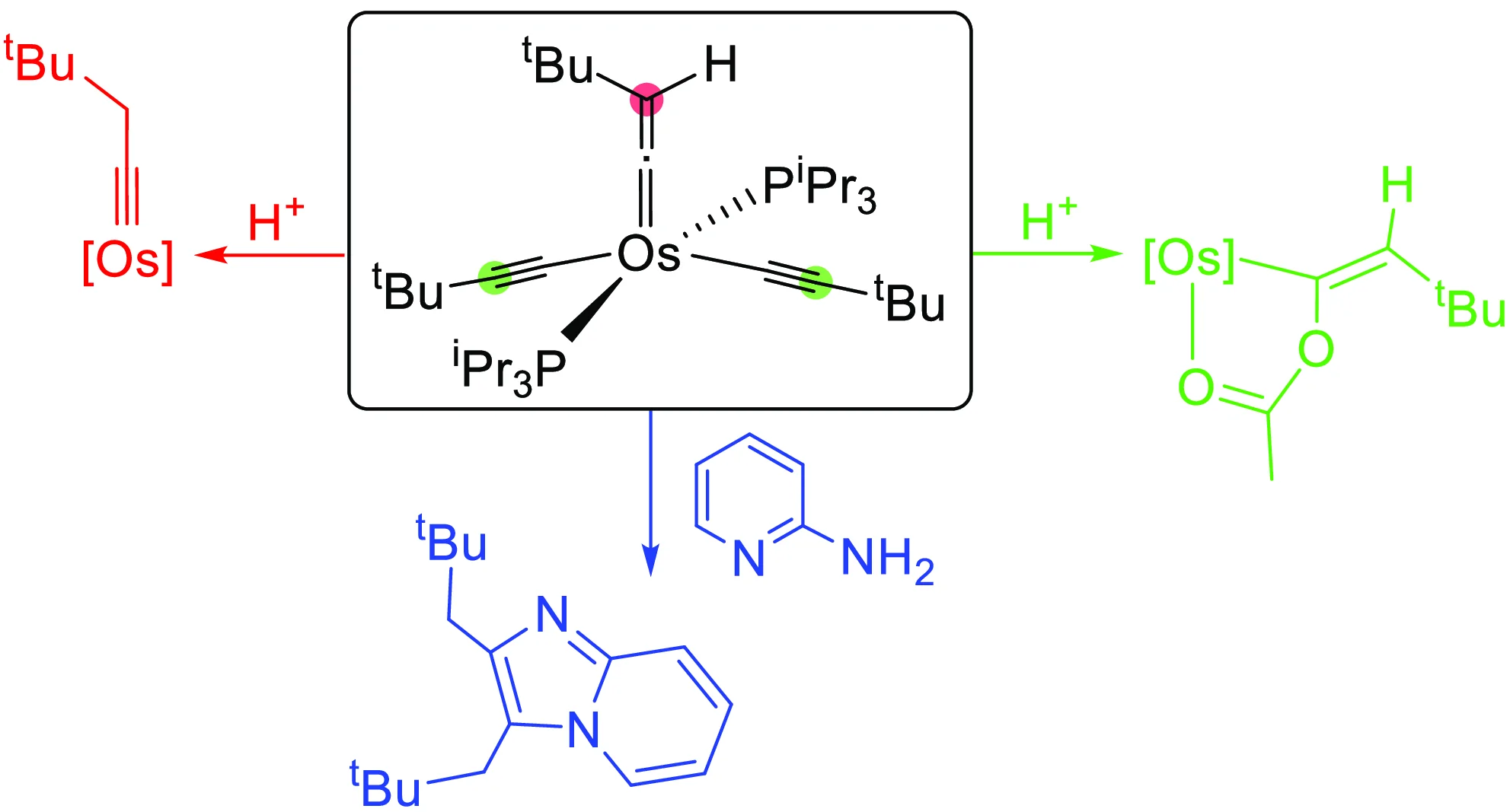

## Introduction

Terminal alkynes are fundamental molecules in organic chemistry and their reactions with transition metal complexes have been a long-standing topic in organometallic chemistry, as they give rise not only to a variety of interesting organic compounds, but also to a rich stoichiometric chemistry.^1^ Their reactions with transition metal hydride complexes are particularly appealing. In general, when the transition-metal compound contains only one hydride ligand, terminal alkynes insert into the M–H bond to give alkenyl derivatives.^2^ Besides alkenyl complexes, the nature of products that can be obtained by reacting terminal alkynes with transition metals rises notably when the number of hydrides coordinated to the metal center increases, and alkynyl, carbene, vinylidene and carbyne derivatives can be obtained.^1^ Behind this wide variety of organometallic products there are several factors, among them the electronic nature of the starting complex and the alkyne precursor. Thus, while phenylacetylene reacts with RuH_2_Cl_2_(P^i^Pr_3_)_2_ to give a 10:1 mixture of the vinylidene and carbene derivatives RuCl_2_(=C=CHPh)­(P^i^Pr_3_)_2_ and RuCl_2_(=CHCH_2_Ph)­(P^i^Pr_3_)_2_,^3^ its reaction with OsH_2_Cl_2_(P^i^Pr_3_)_2_ affords the carbyne complex OsHCl_2_(≡CCH_2_Ph)­(P^i^Pr_3_)_2_ (Scheme 1a).^4^ The reasons behind this striking difference can be derived from the fact that osmium is more reducing than ruthenium and prefers coordination saturation and redox isomers with more metal–carbon bonds.^5^ An illustrative example of the influence of the alkyne precursor can be found in the reactivity of the hydride-osmium­(IV) complex [OsH_2_(κ^2^-O_2_CCH_3_)­(H_2_O)­(P^i^Pr_3_)_2_]­BF_4_ with alkynes.^6^ Thus, while with phenylacetylene affords the hydride-osmacyclopropene derivative [OsH­{=C­(Ph)­CH_2_}­(κ^2^-O_2_CCH_3_)­(P^i^Pr_3_)_2_]­BF_4_, the reaction wit *t*-butylacetylene gives hydride-carbyne complex [OsH­(κ^2^-O_2_CCH_3_)­(≡CCH_2_^t^Bu)­(P^i^Pr_3_)_2_]­BF_4_ (Scheme 1b). These findings were in accordance with the theoretical calculations carried out, which show that the relative stabilities of the metallacyclopropene and carbyne structures are determined by the substituent of the alkyne, being the carbyne thermodynamically less stable than the metallacyclopropene when the substituent is a phenyl moiety, whereas the contrary is found for a *t*-butyl group.

**Scheme 1:**
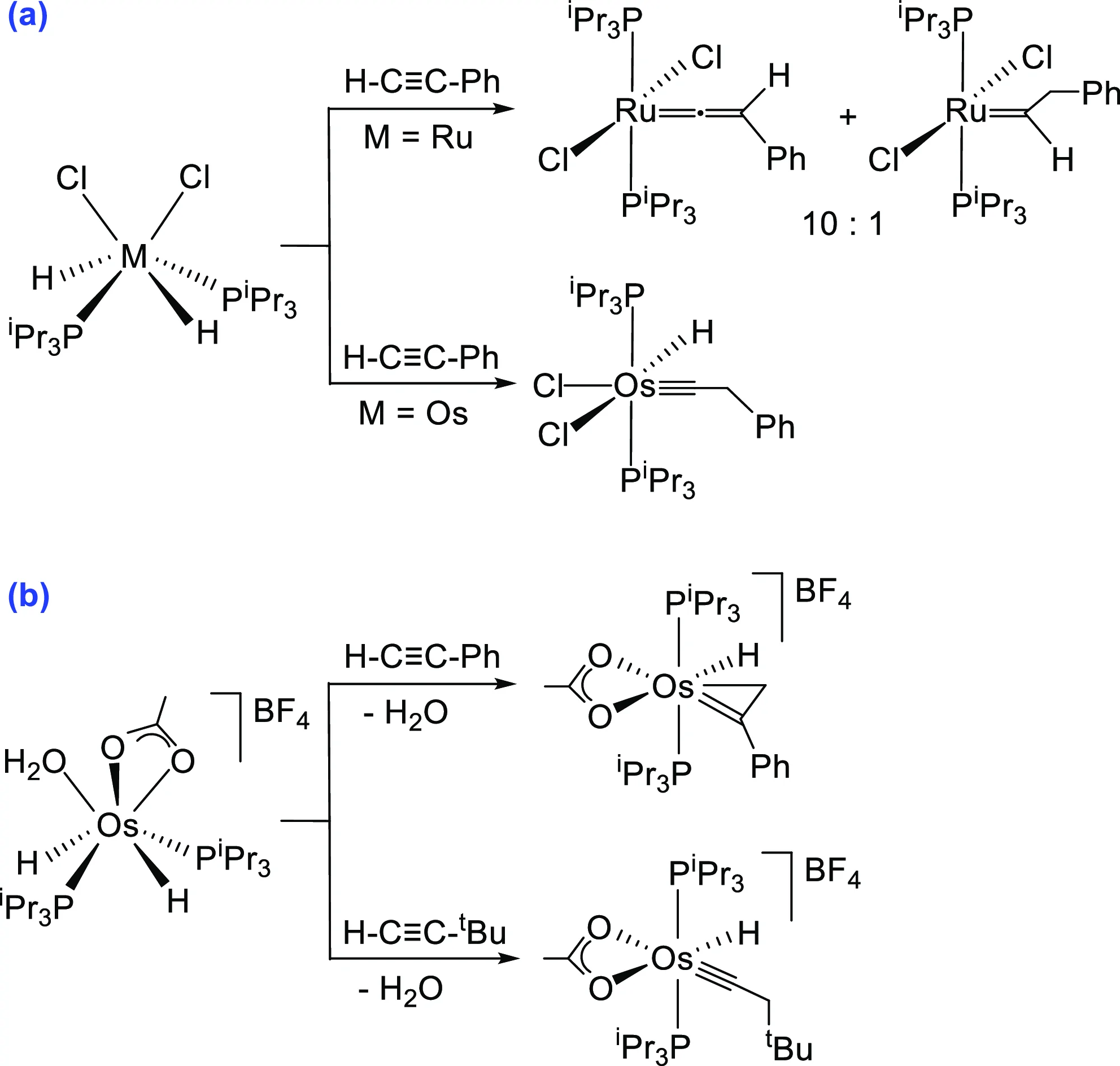
Representative Examples of Metal- (a) and Alkyne-Dependent (b) Reactivity Patterns

The increase of the number of hydride ligands allows as well for an increase in the number of terminal alkynes that have access to the metal center.^7^ Thus, we have previously reported that the hexahydride complex OsH_6_(P^i^Pr_3_)_2_ (**1**) promotes the head-to-head reductive dimerization of phenylacetylenes to afford the 1,4-dibranched-butenediyl derivatives OsH_2_[η^4^-(C_4_H_4_R_2_)]­(P^i^Pr_3_)_2_ (R = C_6_H_5_, C_6_H_4_-*p*-CF_3_, C_6_H_4_-*p*-NMe_2_) (Scheme 2).^8^ The reaction takes place via the insertion of two alkyne molecules into the metal-hydride bonds of the tetrahydride intermediate generated by reductive elimination of dihydrogen from **1**. The insertions give unsaturated bis­(alkenyl)-Os­(II)-(dihydrogen) intermediates, which upon alkenyl coupling and dihydrogen splitting, lead to the observed derivatives.

**Scheme 2:**
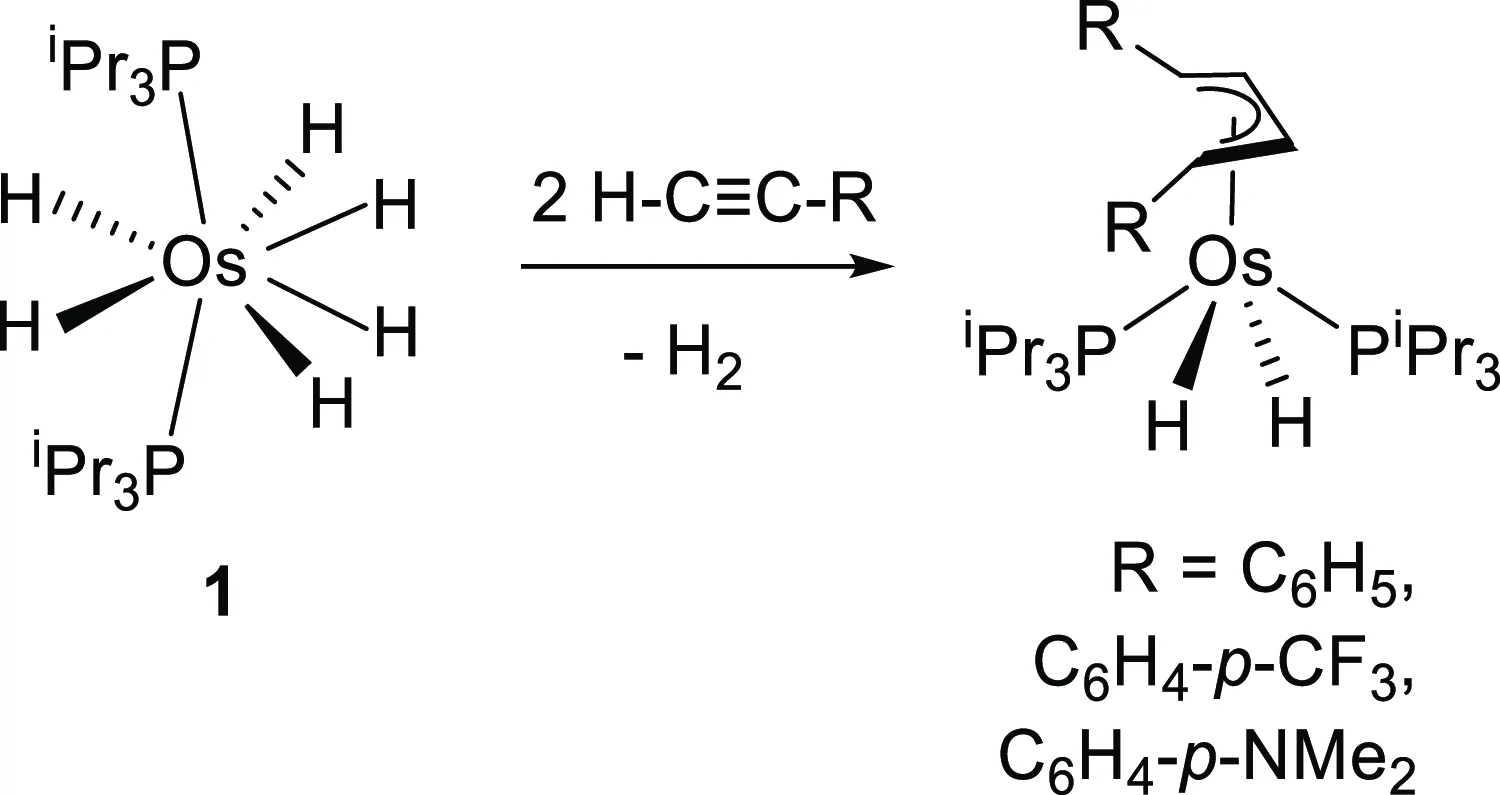
Reactions of 1 with Phenylacetylenes

Now we have found that this head-to-head reductive dimerization reaction does not operate when the substituent of the alkyne is a *t*-butyl group instead of an aromatic one. The reaction of hexahydride **1** with *t*-butylacetylene gives an unusual bis­(alkynyl)-vinylidene derivative, which constitutes a unique platform for the study of competitive reactions toward electrophiles. The outcomes of these studies are reported herein, along with those of its reaction with 2-aminopyridine, which unexpectedly furnishes a 2,3-disubstituted imidazo­[1,2-*a*]­pyridine derivative.

## Results and Discussion

### Reaction of OsH_6_(P^i^Pr_3_)_2_ (**1**) with *t*-Butylacetylene

Treatment of solutions of hexahydride **1** in toluene with 4 equiv of *t*-butylacetylene, at 45 °C, for 14 h leads to the bis­(alkynyl)-vinylidene-osmium­(II) derivative Os­(C≡C^t^Bu)_2_(=C=CH^t^Bu)­(P^i^Pr_3_)_2_ (**2**), which was isolated as a dark purple solid in 77% yield (Scheme 3).

**Scheme 3:**
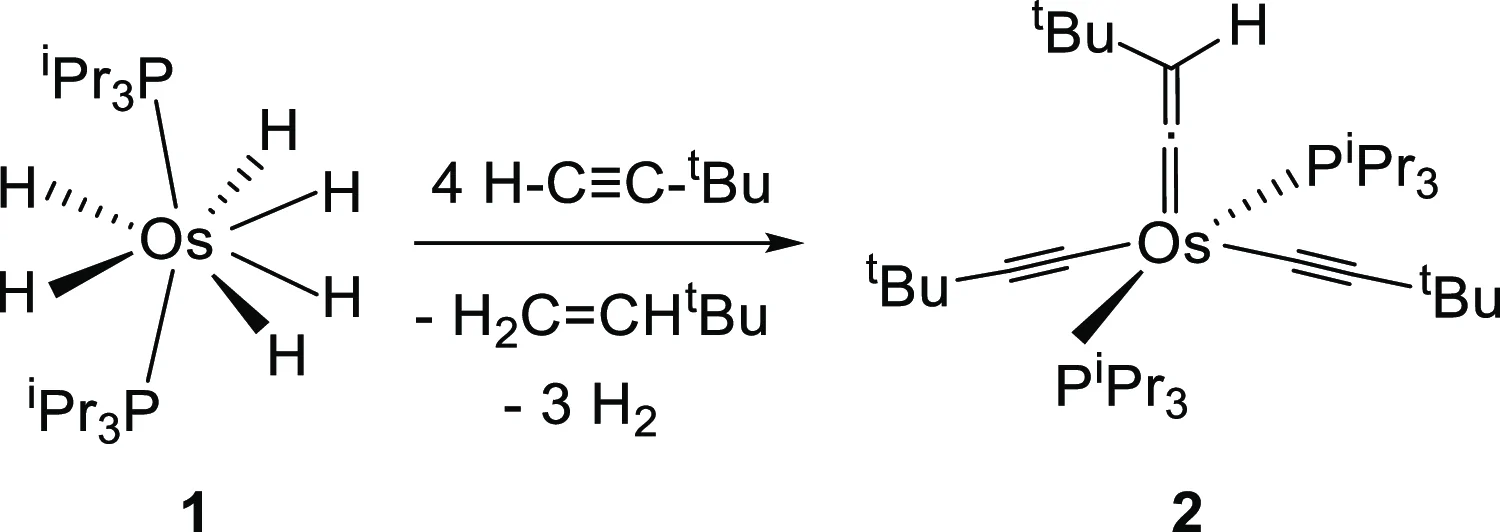
Reaction of 1 with *t*-Butylacetylene

Complex **2** was characterized by X-ray diffraction analysis and Figure 1 shows a view of the structure. The coordination polyhedron that best describes the geometry of this complex is a very distorted square-based pyramid with the vinylidene ligand occupying the axial position and the phosphorus atoms of the phosphines and the alkynyl ligands forming the base. The alkynyl ligands are mutually *trans* disposed, forming an angle (C(7)–Os(1)–C(13) = 143.5(6)°) that strongly deviates from the ideal value of 180° due to the high steric requirement of the *t*-butyl groups. The vinylidene ligand coordinates to the metal center in an almost linear fashion (Os–C(1)–C(2) = 173.2(11)°), while the distances Os–C(1) and C(1)–C(2) of 1.834(15) and 1.32(2) Å, respectively, fit well with those previously reported for other osmium-vinylidene derivatives.^9^ The osmium-alkynyl bond distances Os–C(7) and Os–C(13) of 1.967(11) and 2.012(12), respectively, are consistent with an Os–C­(sp) single bond.^10^

**Figure 1:**
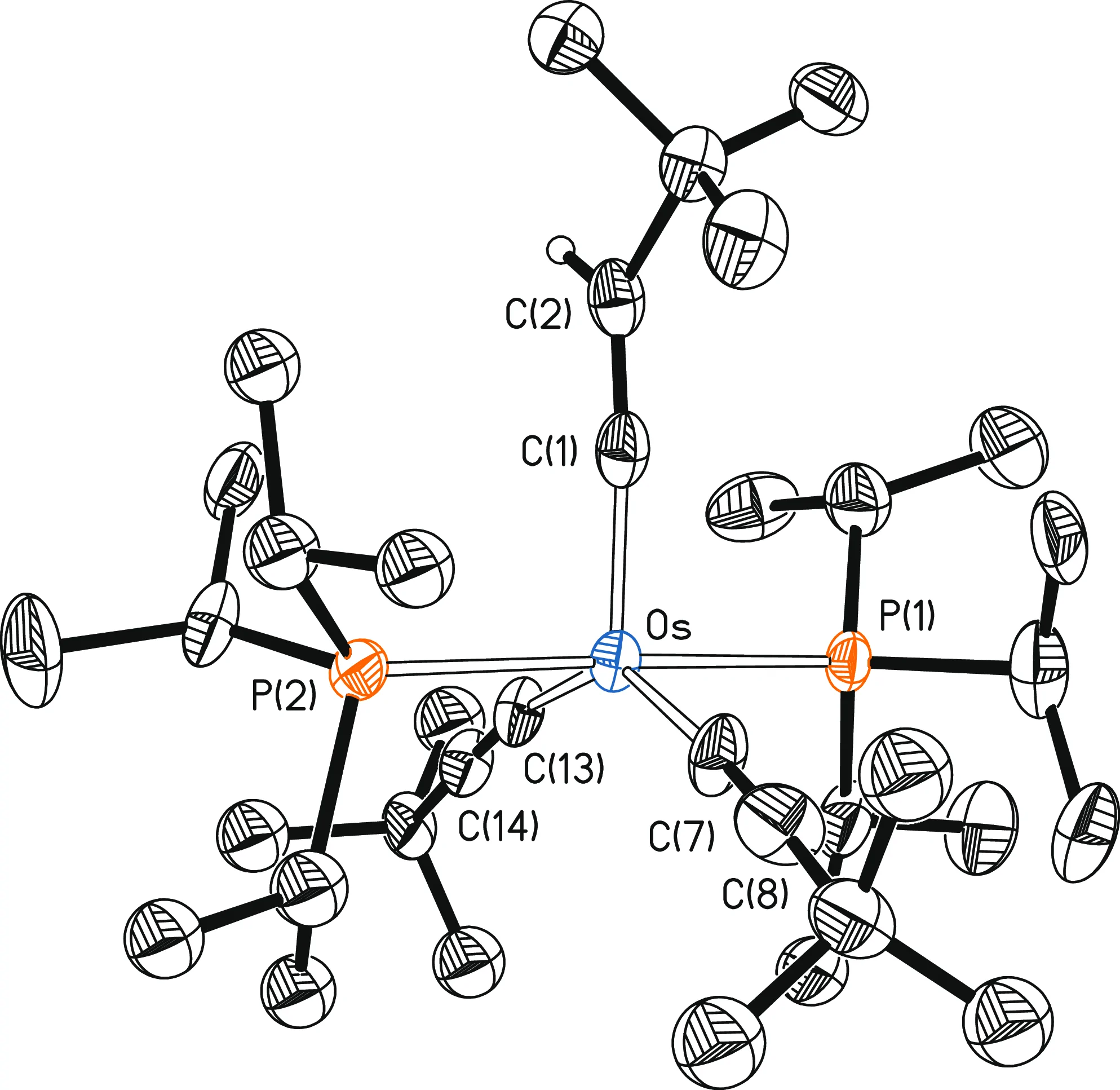
Molecular diagram of complex **2** (displacement ellipsoids shown at 30% probability). All hydrogen atoms (except the vinylidene C_β_ proton) are omitted for clarity. Selected bond distances (Å) and angles (^o^): Os–P(1) = 2.399(3), Os–P(2) = 2.391(3), Os–C(1) = 1.834(15), Os–C(7) = 1.967(11), Os–C(13) = 2.012(12), C(1)–C(2) = 1.32(2), C(7)–C(8) = 1.256(18), C(13)–C(14) = 1.222(18); P(1)–Os–P(2) = 175.61(14), Os–C(1)–C(2) = 173.2(11), C(1)–Os–C(7) = 109.2(6), C(1)–Os–C(13) = 107.3(6), C(7)–Os–C(13) = 143.5(6).

In solution at room temperature the vinylidene ligand rotates, making both alkynyl groups equivalent, as reveals the ^13^C­{^1^H} NMR spectrum recorded in benzene-*d*_6_, that shows a broad signal at 146.9 ppm assigned to the C_α_ carbon atoms and a singlet at 112.6 ppm attibuted to the C_β_ carbon atoms of these ligands, while the C_α_ and C_β_ atoms of the vinylidene moiety appear as triplets at 268.0 ppm (^2^ *J*_C–P_ = 10.3 Hz) and 117.0 ppm (^3^ *J*_C–P_ = 4.6 Hz), respectively. In the ^1^H NMR spectrum the resonance assigned to the C_β_ proton of the vinylidene appears at 0.61 ppm as a triplet (^4^ *J*_H–P_ = 3.4 Hz).

Complexes contaning two alkynyl groups and a vinylidene moiety have been proposed as intermediates in the stereocontrolled dimerization of *t*-butylacetylene to (*Z*)-l,4-di-*t*-butylbutatriene,^11^ as well as in the dimerization of aryl acetylenes to afford (*Z*)-1,3-enynes,^7^ ^d^ but have been isolated only in a few cases.^7^ ^c,7e^ We have observed that complex **2** (toluene, 5 mol %) is a modest catalyst for the dimerization of *t*-butylacetylene, and that the products obtained depend on the reaction temperature. Thus, when the reaction is carried out at room temperature, it quantitatively and selectively produces (*Z*)-1,4-di-*t*-butylbutatriene after 3 days, while rising the temperature to 50 °C leads to a mixture 70:30 of the cumulene and of the (*Z*)-1,3-enyne isomer after 11 h. The enyne is the result of the isomerization of the cumulene. The heating (80 °C, 5 h) of the catalytic solution obtained once the butatriene is formed leads to a mixture butatriene: enyne 30:70. In addition, the stereochemistry of the enyne suggests than that of the cumulene is also *Z*, since the discrimination between *Z* and *E* cannot be done since both isomers display almost identical ^1^H NMR chemical shifts.^12^

Both alkynyl and vinylidene complexes are cornerstones in organometallic chemistry and have well-established reactivity patterns. It is known that the coordination of an alkynyl group to a metal center transfers the nucleophilicity from the C_α_ to the C_β_ carbon atom, and that vinylidene ligands are electron defficient at C_α_, while their C_β_ carbon atoms are electron rich.^13^ Therefore, both alkynyl and vinylidene groups are susceptible to react with electrophiles and this makes complex **2** a unique scenario for studying the competition between them.

The study of the Natural Bond Orbital (NBO) charges on the C_α_ and C_β_ carbon atoms of the vinylidene and alkynyl ligands of complex **2** (Figure 2a, optimized by DFT at level B3LYP-D3//SDD­(F)-631G**) reveals that the carbon atom with the greatest negative charge is the C_β_ carbon atom of the vinylidene group, followed by the C_α_ and C_β_ carbon atoms of the alkynyl moieties. Accordingly with the NBO charges, the analysis of the orbitals of complex **2** (Figure 2b) reveals that the HOMO is mainly located on the vinylidene ligand (37% on the C_β_ carbon atom), while the HOMO–1 lies on the alkynyl groups (15% and 18% on each C_β_ carbon atom). Therefore, both predict that an electrophilic attack to this complex will take place at the C_β_ carbon atom of the vinylidene ligand.

**Figure 2:**
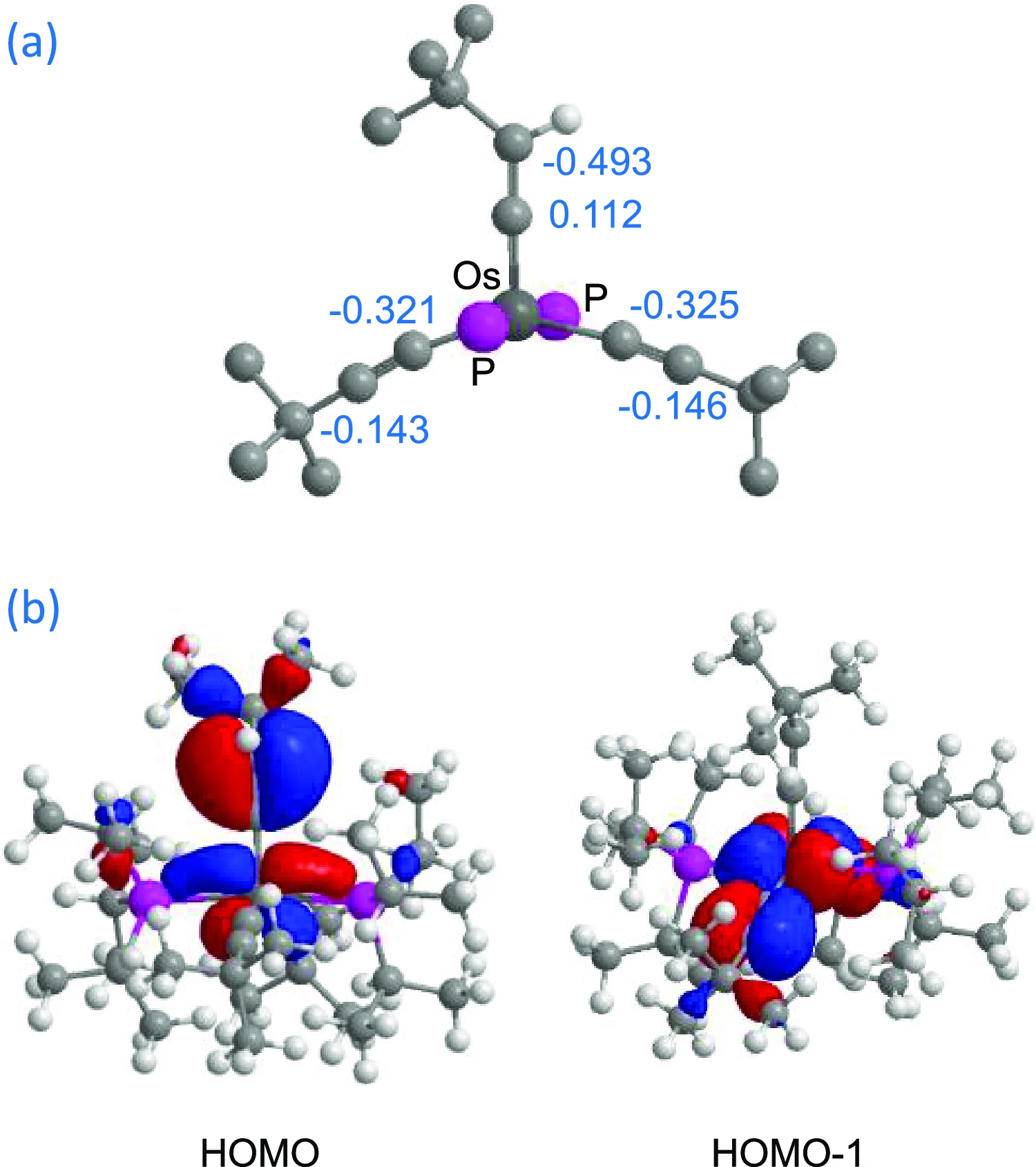
(a) NBO charges on the C_α_ and C_β_ carbon atoms of the vinylidene and alkynyl ligands of complex **2**. (b) Computed HOMO and HOMO–1 of complex **2** (isosurface value 0.03 au).

### Reactions of Bis­(alkynyl)-vinylidene 2 with Acids

In accordance with the predictions, complex **2** reacts with trifluoromethanesulfonic acid in dichloromethane-*d*_2_ at 233 K to afford the bis­(alkynyl)-carbyne salt [Os­(C≡C^t^Bu)_2_(≡CCH_2_^t^Bu)­(P^i^Pr_3_)_2_]­OTf (**3**) as a result of the electrophilic attack of the proton to the C_β_ carbon atom of the vinylidene ligand of **2** (Scheme 4), confirmed by the reaction of **2** with the deuterated acid. Figure 3 shows a molecular diagram of the cation of this species. The geometry around the osmium center is similar to that of complex **2**, with the carbyne moiety in the position of the vinylidene ligand. In salt **3**, the osmium-alkynyl bond lengths Os–C(7) and Os–C(13), 2.028(4) and 2.042(4) Å, respectively, are similar to the corresponding parameters of **2**, while the angle between the alkynyl ligands (C(7)–Os–C(13) = 153.03(16)°) is around 10° larger than the related one in **2**. The opening of this angle is accompanied by a decrease of almost 20° in the P(1)–Os–P(2) angle, which can be derived from the shortening of the Os–C(1) distance (1.703(4) Å), which brings the *t*-butyl group attached to C(2) closer to the metallic center and forces the remaining ligands to rearrange in order to decrease the steric hindrance. The value of the Os–C(1) distance, as well as the Os–C(1)–C(2) angle (175.1(3)°), are in accordance with those formerly reported for osmium-carbyne derivatives.^4,14^ The presence of a carbyne ligand in **3** is further supported by the ^1^H and ^13^C­{^1^H} NMR spectra of this salt in dichloromethane-*d*_2_ at 233 K. The ^1^H NMR spectrum contains a broad signal at 2.19 ppm assigned to the CH_2_ group, whereas the ^13^C­{^1^H} NMR spectrum shows a singlet at 282.8 ppm for the C­(sp) carbon atom and a singlet at 66.1 ppm correspongding to the CH_2_ group.

**Figure 3:**
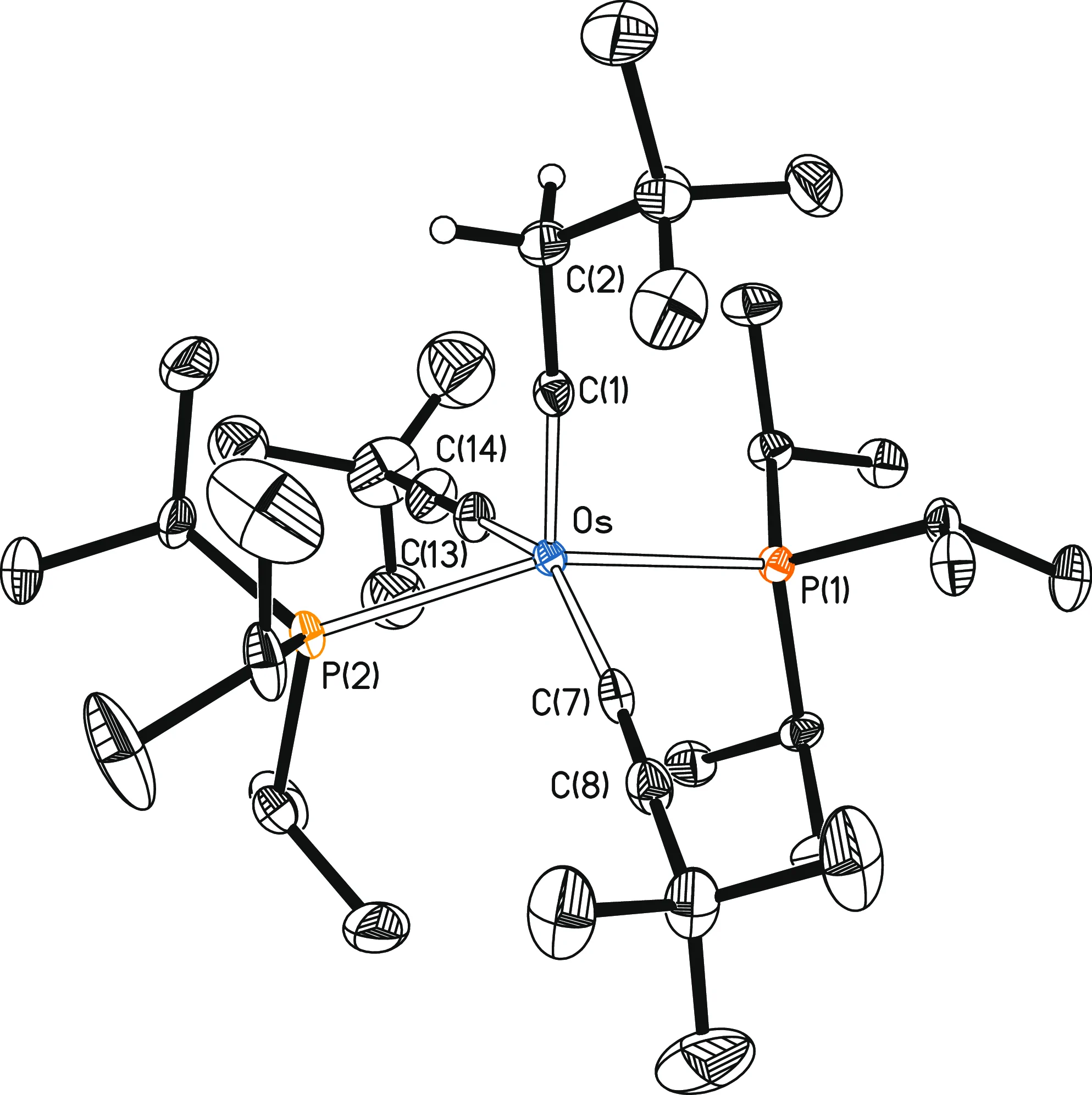
Molecular diagram of the cation of complex **3** (displacement ellipsoids shown at 50% probability). All hydrogen atoms (except those of the carbyne C_β_ protons) are omitted for clarity. Selected bond distances (Å) and angles (^o^): Os–P(1) = 2.4376(9), Os–P(2) = 2.4303(9), Os–C(1) = 1.703(4), Os–C(7) = 2.028(4), Os–C(13) = 2.042(4), C(1)–C(2) = 1.472(5), C(7)–C(8) = 1.213(5), C(13)–C(14) = 1.195(6); P(1)–Os–P(2) = 157.80(3), C(1)–Os–C(7) = 103.28(16), C(1)–Os–C(13) = 103.42(17), C(7)–Os–C(13) = 153.03(16), Os–C(1)–C(2) = 175.1(3).

**Scheme 4:**
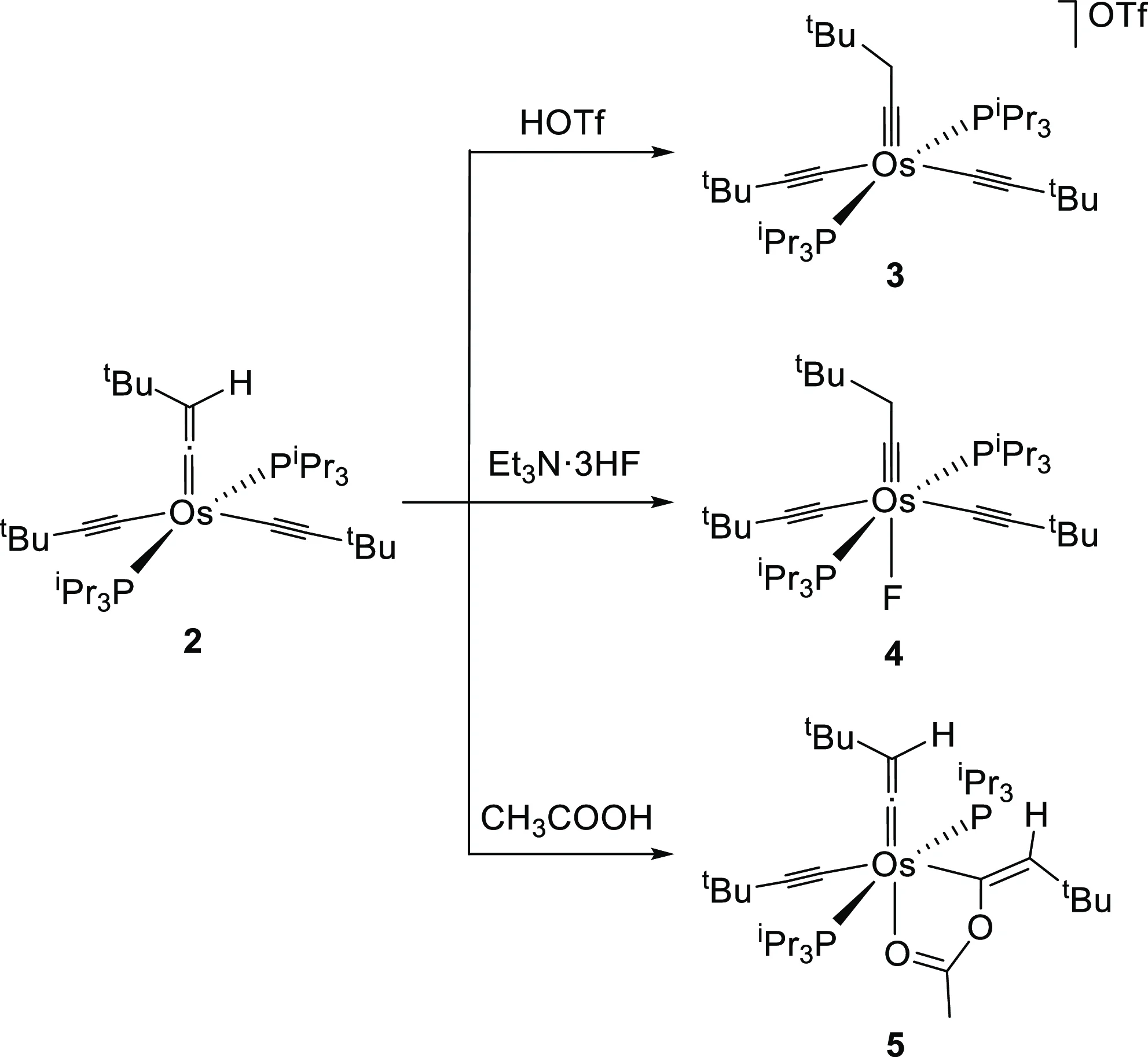
Reaction of Bis­(alkynyl)-Vinylidene 2 with Acids

The reaction of complex **2** with an acid whose anion has coordinating ability such as Et_3_N·3HF leads to the saturated bis­(alkynyl)-carbyne derivative OsF­(C≡C^t^Bu)_2_(≡CCH_2_^t^Bu)­(P^i^Pr_3_)_2_ (**4**). Its solid state structure (Figure 4) confirms the coordination of the fluoride anion to the metal center. Compared to the structure of salt **3**, the coordination of the anion opens around 14° the angle C(7)–Os(1)–C(13) (167.3(2)°) and enlarges around 0.04 Å the osmium-alkynyl bond lengths Os–C(7) and Os–C(13), 2.083(6), 2.088(6) Å, and 2.084(6), 2.089(6) Å, respectively. Both the Os–C(1) distances (1.761(5), 1.749(5) Å) and the Os–C(1)–C(2) angles (174.4(5), 177.1(5)°), are similar to the related parameters in complex **3**. The coordination of the fluoride anion is also evident in the ^13^C­{^1^H} NMR spectrum recorded in benzene-*d*_6_, where the resonances assigned to the C­(sp) and C­(sp^3^) carbon atoms of the carbyne moiety appear as a doublet of triplets at 283.6 ppm (^2^ *J*_C–F_ = 127.7 Hz, ^2^ *J*_C–P_ = 10.2 Hz) and a doublet at 65.7 ppm (^3^ *J*_C–F_ = 12.1 Hz), respectively, and those of the C_α_(sp) and C_β_(sp) carbon atoms of the alkynyl groups which appear as a broad signal at 121.4 ppm and as a doublet of triplets at 89.6 ppm (^2^ *J*_C–F_ = 19.2 Hz, ^2^ *J*_C–P_ = 12.1 Hz), respectively.

**Figure 4:**
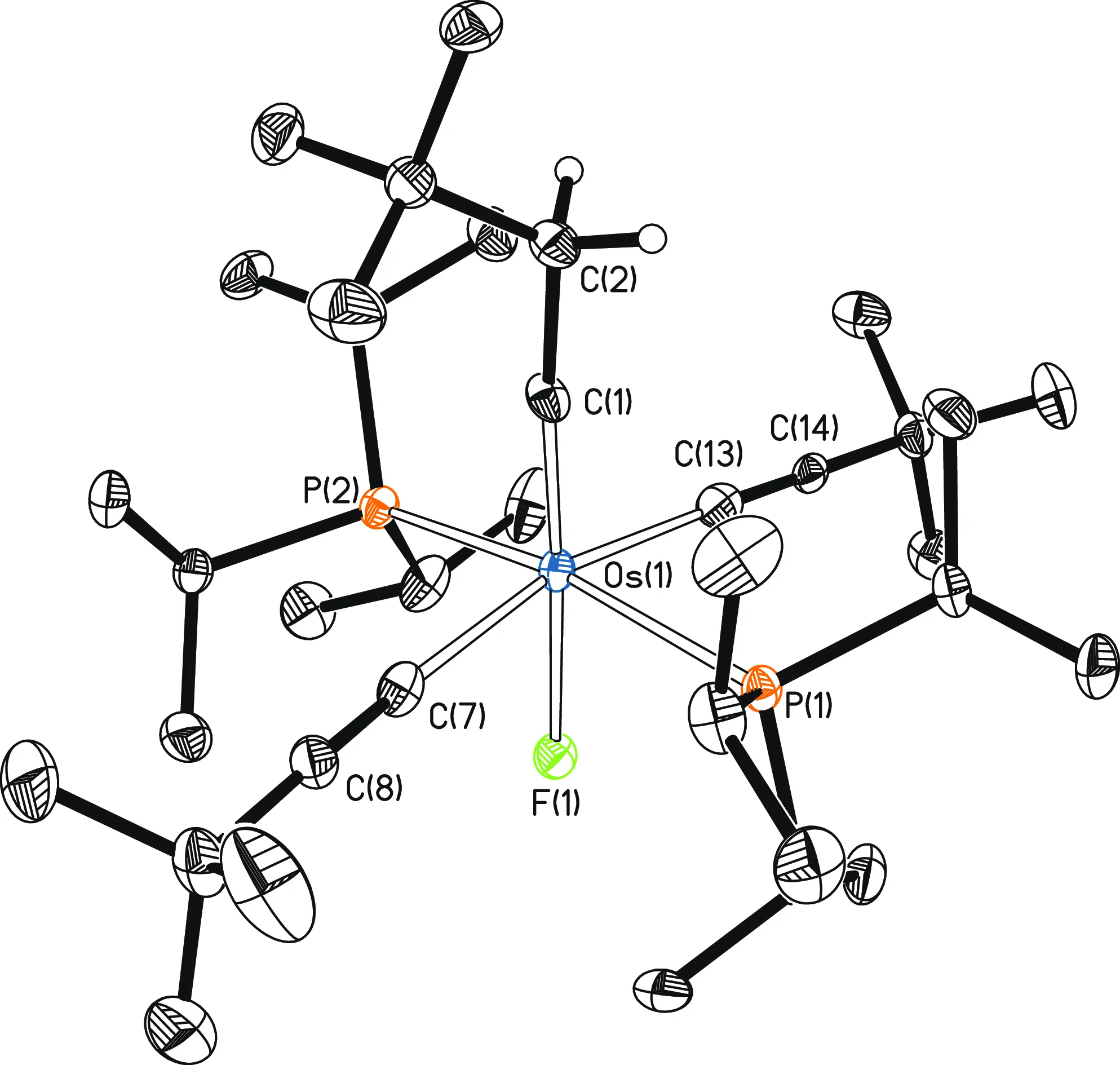
Molecular diagram of one of the two independent molecules of complex **4** (displacement ellipsoids shown at 50% probability). All hydrogen atoms (except the carbyne C_β_ protons) are omitted for clarity. Selected bond distances (Å) and angles (^o^): Os(1)–P(1) = 2.4484(13), 2.4309(14), Os(1)–P(2) = 2.4330(14), 2.4455(14), Os(1)–F(1) 2.075(3), 2.073(3), Os(1)–C(1) 1.761(5), 1.749(5), Os(1)–C(7) 2.083(6), 2.088(6), Os(1)–C(13) 2.084(6), 2.089(6), C(1)–C(2) 1.454(7), 1.462(7), C(7)–C(8) 1.192(8), 1.205(8), C(13)–C(14) 1.201(8), 1.210(8); P(1)–Os(1)–P(2) = 167.69(5), 168.23(5), C(1)–Os(1)–F(1) = 176.0(2), 175.72(18), C(7)–Os(1)–C(13) = 167.3(2), 165.8(2), C(1)–Os(1)–C(7) = 95.3(2), 93.9(2), C(1)–Os(1)–C(13) = 96.0(2), 98.4(2), Os(1)–C(7)–C(8) = 174.7(5), 173.7(5), Os(1)–C(13)–C(14) = 178.1(5), 173.7(5), Os–C(1)–C(2) = 174.4(5), 177.1(5).

Treatment of **2** with acetic acid in pentane does not lead to the expected bis­(alkynyl)-carbyne complex related to derivative **4**, and the alkynyl-vinylidene-vinyl ester derivative Os­(C≡C^t^Bu)­{κ^2^-*O,C*-OC­(CH_3_)­OC=CH^t^Bu}­(=C=CH^t^Bu)­(P^i^Pr_3_)_2_ (**5**, Scheme 4) is formed instead. The reaction of **2** with acetic acid in dichloromethane also affords complex **5**, although it proceeds more cleanly in pentane. Complex **5** was isolated as a yellow solid in 84% yield and Figure 5 shows the molecular diagram of one of the two crystallographically independent, but chemically equivalent, molecules of **5** found in the unit cell. The X-ray structure confirms the presence of three different C-donor ligands in the coordination sphere of the osmium atom. The values of the Os(1)–C(9) (2.071(3), 2.062(3) Å), Os(1)–C(15) (1.822(3), 1.820(3) Å), Os(1)–C(1) (2.102(3), 2.108(3) Å) are in agreement with those previously reported for osmium-alkynyl,^7e,10a,15^ osmium-vinylidene^9^ and osmium-vinyl^7b,16^ derivatives, respectively. Further support for the existence of these ligands arises from the ^1^H and ^13^C­{^1^H} NMR spectra of the complex in benzene-*d*_6_ at room temperature. The ^1^H NMR spectrum contains a broad signal at 4.51 ppm and a triplet (^4^ *J*_H–P_ = 3.1 Hz) at 0.34 ppm assigned to the vinylic and vinylidene protons, respectively. The resonances assigned to the three different ligands in the ^13^C­{^1^H} NMR spectrum appear as singlets at 146.9 (C_α_) and 113.7 ppm (C_β_) (alkynyl), triplets at 285.4 (^2^ *J*_C–P_ = 11.2 Hz; C_α_) and 116.9 ppm (^3^ *J*_C–P_ = 3.5 Hz; C_β_) (vinylidene), and a triplet at 172.6 (^2^ *J*_C–P_ = 11.6 Hz; C_α_) and a singlet at 130.5 ppm (C_β_) (vinyl).

**Figure 5:**
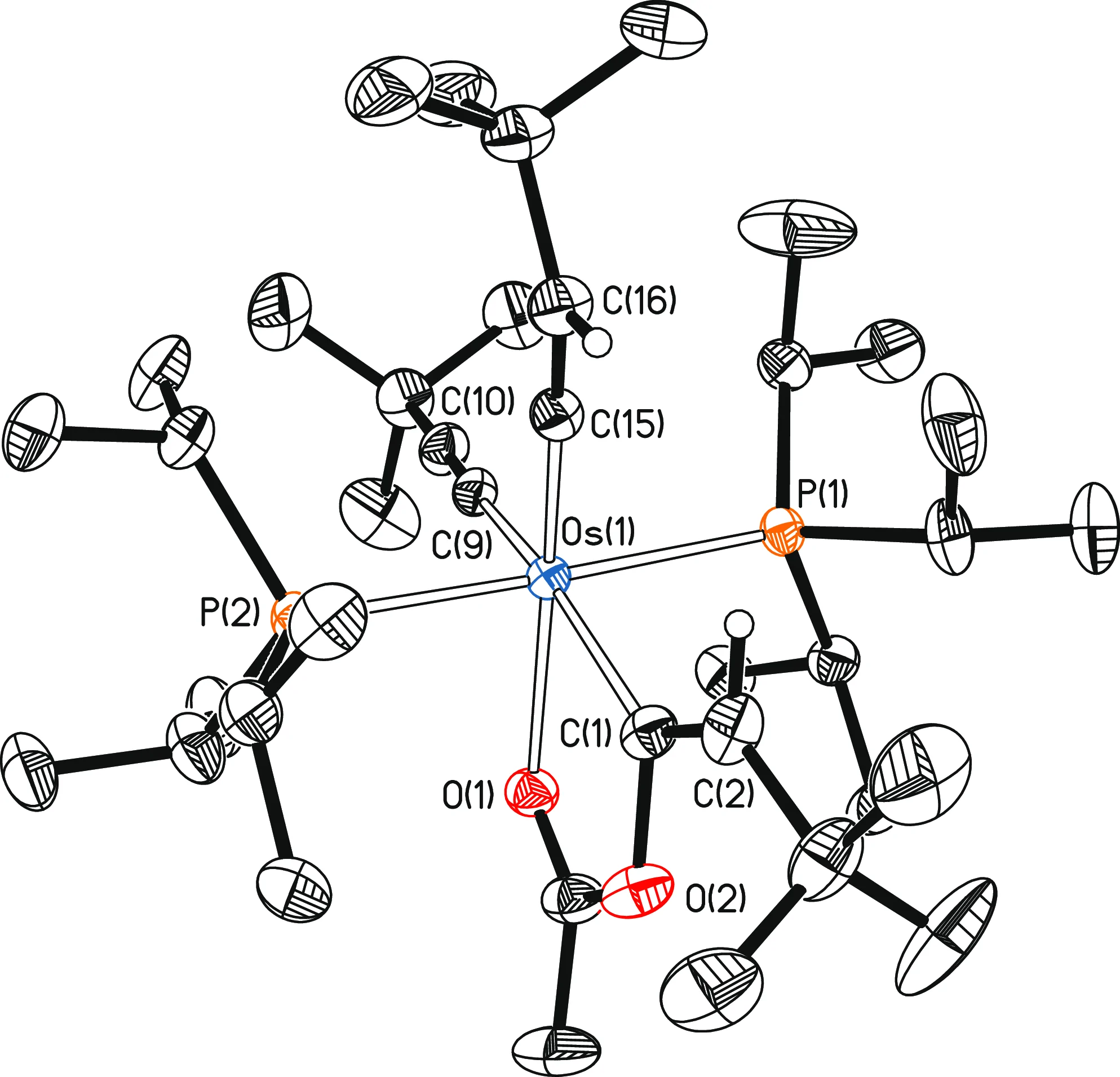
Molecular diagram of one of the two independent molecules of complex **5** (displacement ellipsoids shown at 50% probability). All hydrogen atoms (except the C_β_ protons of the vinylidene and vinyl moieties) are omitted for clarity. Selected bond distances (Å) and angles (^o^): Os(1)–P(1) = 2.4185(7), 2.4159(7), Os(1)–P(2) = 2.4051(6), 2.4122(7), Os(1)–O(1) = 2.2292(18), 2.2226(18), Os(1)–C(1) = 2.102(3), 2.108(3), Os(1)–C(9) = 2.071(3), 2.062(3), Os(1)–C(15) = 1.822(3), 1.820(3), C(1)–C(2) = 1.328(4), 1.341(4), C(9)–C(10) = 1.199(4), 1.204(4), C(15)–C(16) = 1.333(4), 1.344(4); P(1)–Os(1)–P(2) = 175.01(2), 174.86(2), C(15)–Os(1)–O(1) = 167.87(10), 169.69(11), C(9)–Os(1)–C(1) = 160.49(10), 160.89(10), C(1)–Os(1)–O(1) = 74.93(9), 74.88(9).

To determine why complex **5** is formed instead of the analog of **4** we have performed DFT calculations. The free energy variations (Δ*G*) were calculated at 298.15 K, 1 atm, and using dichloromethane and pentane as solvents. In dichloromethane, the electrophilic attack of H^+^ to the C_β_ carbon atom of the vinylidene ligand would afford the bis­(alkynyl)-carbyne Os­{κ^1^-OC­(=O)­CH_3_}­(C≡C^t^Bu)_2_(≡CCH_2_^t^Bu)­(P^i^Pr_3_)_2_ (**4**_**Ac**_; Figure 6, red pathway). This process is energetically feasible (13.7 kcal mol^–1^), but the resulting **4**_**Ac**_ is located 8.9 kcal mol^–1^ above **2**. This is consistent with the fact that this compound is not observed experimentally. Unlike **4**_**Ac**_, the formation of complex **4** is thermodynamically favorable with respect to **2** + HF (Figure S1). Complex **5** seems to be the result of the electrophilic attack of H^+^ to one of the C_β_ carbon atoms of the alkynyl groups of complex **2** to afford an alkynyl-bis­(vinylidene) intermediate, followed by the nucleophilic attack of the oxygen atom of the carbonyl group of the acetate ligand to the C_α_ carbon atom of the newly formed vinylidene moiety. The process (Figure 6, blue pathway) would start by coordination of the acid to the vacant site of derivative **2**, which would afford the saturated intermediate **A**, located 3.7 kcal mol^–1^ above complex **2**. Then the electrophilic attack of the acidic proton of the coordinated acid to one of the C_β_ carbon atoms of the alkynyl ligands would give the alkynyl-bis­(vinylidene) intermediate **B**. This process takes place via transition state **TS**_**A‑B**_, located 23.5 kcal mol^–1^ over complex **2**. Since in complex **5** the oxygen atom and the proton are *anti* disposed, the vinylidene ligand in intermediate **B** must rotate prior to the nucleophilic attack of the oxygen atom to its C_α_ carbon atom takes place. The barrier for this rotation is of just 3.6 kcal mol^–1^, value similar to those estimated in previous theoretical studies,^17^ and gives intermediate **C**. The nucleophilic attack of the carbonyl oxygen atom of the coordinated acetate ligand to the C_α_ carbon atom of the vinylidene ligand *cis* disposed to it in intermediate **C** would give complex **5**, that is the only organometallic species detected in the reaction of complex **2** with acetic acid.

**Figure 6:**
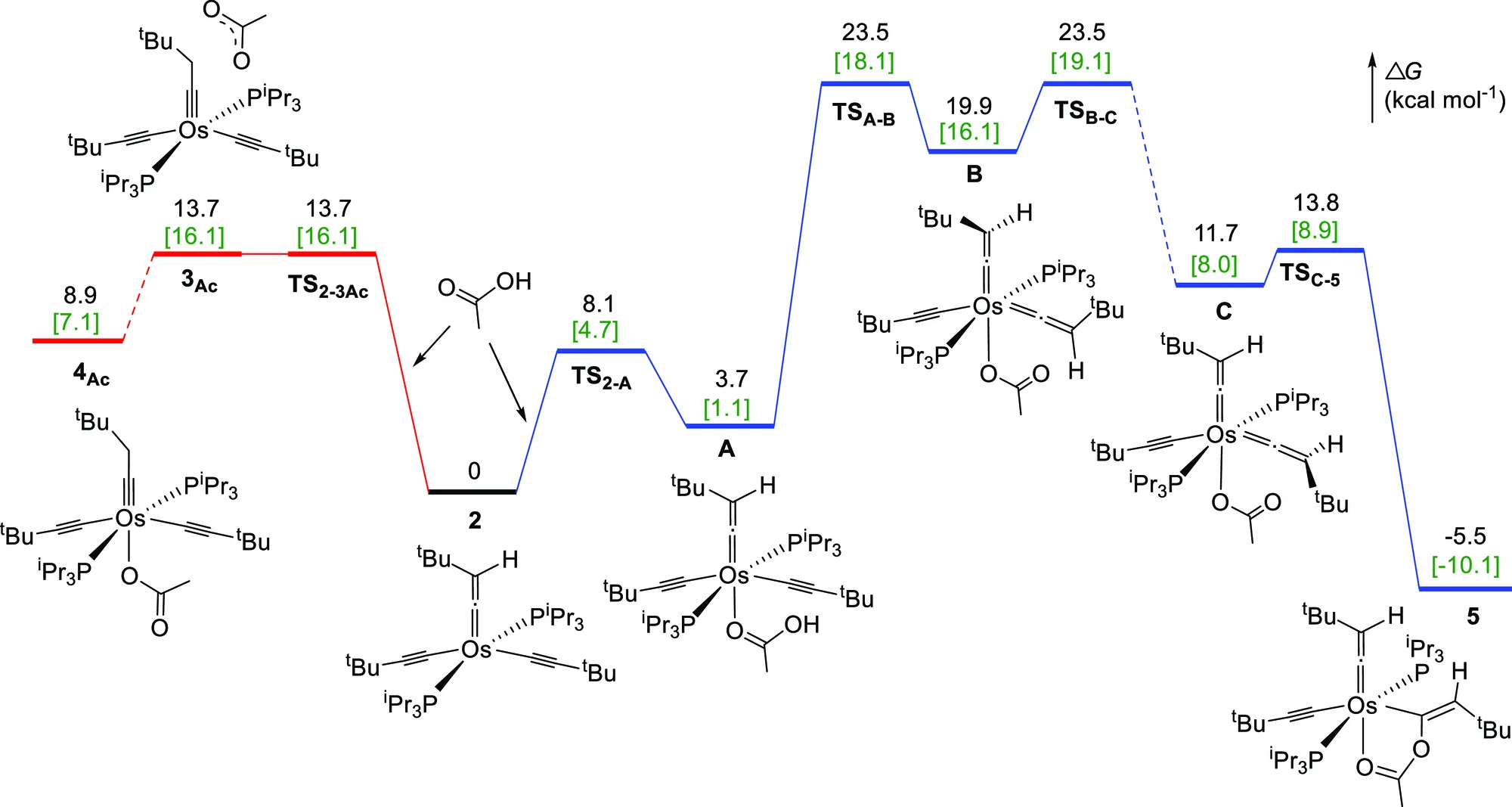
Computed energy profiles (Δ*G* in kcal mol^–1^) for the reaction of **2** with acetic acid in dichloromethane and pentane (values in square brackets). Red pathway: electrophilic attack of H^+^ to the C_β_ carbon atom of the vinylidene. Blue pathway: electrophilic attack of H^+^ to one of the C_β_ carbon atoms of the alkynyl groups.

The reaction profile in pentane is qualitatively similar (Figure 6; values in square brackets). However, a closer analysis reveals that the barrier for the formation of **5** through the electrophilic attack of the acidic proton of the coordinated acid of intermediate **A** to one of the C_β_ carbon atoms of the alkynyl ligands to give the alkynyl-bis­(vinylidene) intermediate **B** is lower in pentane (19.1 kcal mol^–1^) than in dichloromethane (23.5 kcal mol^–1^). In addition, complex **5** is more stabilized relatively to **2** in pentane (Δ*G* = −10.1 kcal mol^–1^) than in dichloromethane (Δ*G* = −5.5 kcal mol^–1^). These results are consistent with the experimental observation that the reaction proceeds more cleanly in pentane.

To gain further insight into this process we carried out deuterium labeled experiments. The ^2^H NMR spectrum of the reaction of complex **2** with acetic acid-*d*_4_ in benzene (Figure S23) shows broad signals of similar intensities at 4.51 and 0.42 ppm, indicating that there is deuterium incorporation at both vinyl and vinylidene C_β_ positions. This observation is accompanied by a decrease in the integral of these signals in the ^1^H NMR spectrum registered in benzene-*d*_6_. This result is in agreement with the theoretical study of the reaction and demonstrates experimentally that, in this case, the protonation can take place in two sites, and that the observed product is that of thermodynamic control.

### Reactions of Bis­(alkynyl)-vinylidene 2 with 2-Aminopyridine

After investigating the reactivity of complex **2** with electrophiles, we became interested in its behavior toward nucleophiles. To this end, 2-aminopyridine was selected as a model nucleophile. Nucleophilic attack of amines at the α-carbon atom of vinylidene ligands is a well-established strategy for the synthesis of Fischer-type transition-metal carbene complexes.^13^

The reaction of **2** with 3 equiv of 2-aminopyridine was carried out in toluene at 80 °C. The ^31^P­{^1^H} NMR spectrum recorded after 14 h revealed the presence of singlets at 19.6 and 54.8 ppm, corresponding to free triisopropylphosphine and its oxide, respectively. In the ^1^H NMR spectrum, besides several broad resonances in the aliphatic region, a number of well-resolved signals were also observed (see Figure S26). Subsequent purification by column chromatography on silica gel (pentane/diethyl ether as eluent) afforded a yellowish oil. Its ^1^H NMR spectrum displays four resonances in the aromatic region and four singlets in the aliphatic one. Definitive structural assignment was achieved by an X-ray diffraction study, performed in crystals obtained upon crystallization of the oil in diethyl ether, which confirmed the formation of 2,3-dineopentylimidazo­[1,2-*a*]­pyridine (**6**; Figure 7). Given the interest of molecules containing the imidazo­[1,2-*a*]­pyridine core,^18^ we investigated if derivative **6** could be prepared using complex **2** as a catalyst. In fact, complex **2** proved to be an effective catalyst for the annulation of 2-aminopyridine with *tert*-butylacetylene, delivering **6** in 85% isolated yield (Scheme 5) under optimized conditions (5 mol %, toluene, 100 °C, 20 h). This reaction provides a new route for the selective synthesis of 2,3-disubstitued imidazo­[1,2-*a*]­pyridine derivatives starting from *tert*-butylacetylene and 2-aminopyridine.

**Figure 7:**
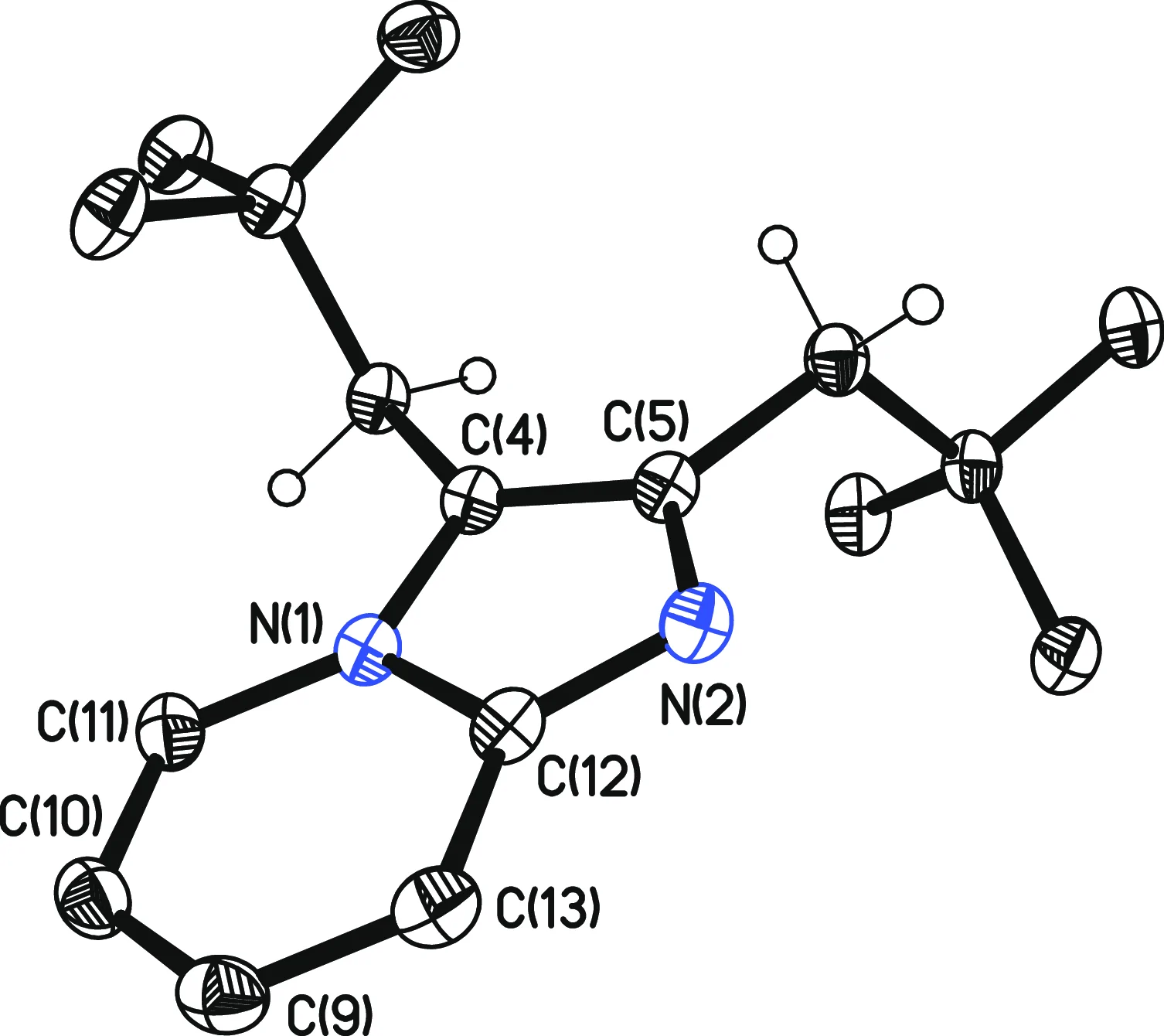
Molecular diagram of **6** (displacement ellipsoids shown at 50% probability). All hydrogen atoms (except the protons of the CH_2_ groups) are omitted for clarity. Selected bond distances (Å): N(1)–C(11) = 1.3804(13), C(11)–C(10) = 1.3594(15), C(10)–C(9) = 1.4216(16), C(9)–C(13) = 1.3647(16), C(13)–C(12) = 1.4178(14), C(12)–N(1) = 1.3979(12), N(1)–C(4) = 1.3940(12), C(4)–C(5) = 1.3829(13), C(5)–N(2) = 1.3813(13), N(2)–C(12) = 1.3288(13).

**Scheme 5:**
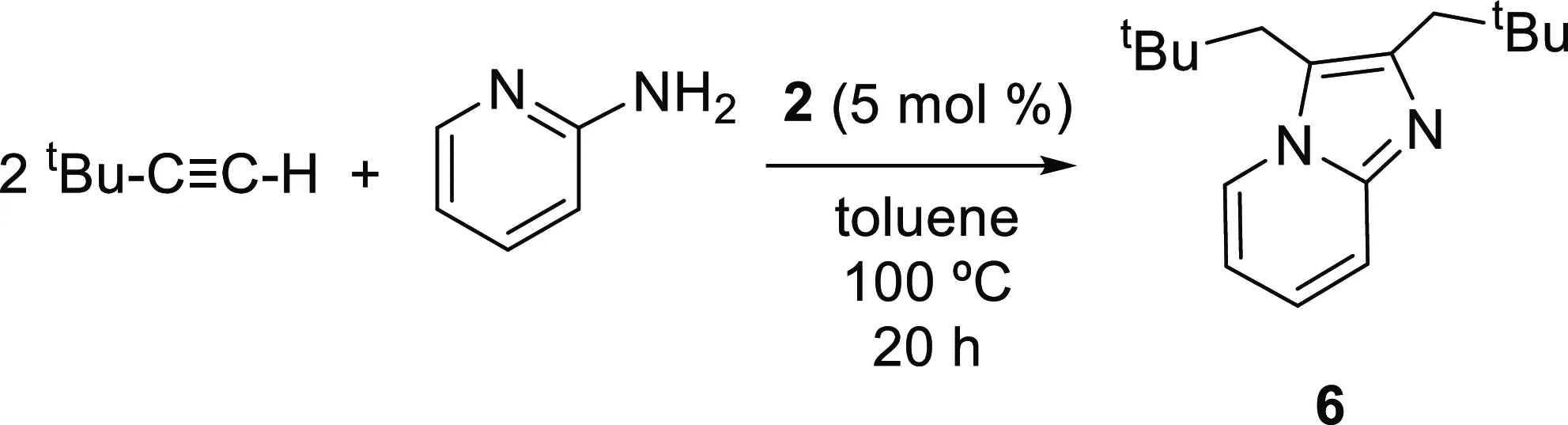
Synthesis of 2,3-Dineopentylimidazo­[1,2-*a*]­pyridine

## Concluding Remarks

This study has revealed that the products of the reactions of hexahydride OsH_6_(P^i^Pr_3_)_2_ with terminal alkynes depend upon the substituent of the alkyne. Thus, while with aromatic substituents 1,4-dibranched-butenediyl derivatives OsH_2_[η^4^-(C_4_H_4_R_2_)]­(P^i^Pr_3_)_2_ (R = C_6_H_5_, C_6_H_4_-*p*-CF_3_, C_6_H_4_-*p*-NMe_2_) are obtained, the reaction with *t*-butylacetylene leads to the bis­(alkynyl)-vinylidene Os­(C≡C^t^Bu)_2_(=C=CH^t^Bu)­(P^i^Pr_3_)_2_.

The analysis of the NBO charges on derivative Os­(C≡C^t^Bu)_2_(=C=CH^t^Bu)­(P^i^Pr_3_)_2_ has revealed that the C_β_ carbon atom of the vinylidene moiety is the most nucleophilic and, accordingly, the reactions with HOTf and Et_3_N·3HF lead to bis­(alkynyl)-carbyne derivatives. However, the addition of acetic acid gives an alkynyl-vinylidene-vinyl ester derivative. The DFT calculations show that, in this case, while the bis­(alkynyl)-carbyne complex is the product of kinetic control, that of addition of H^+^ to one of the C_β_ carbon atoms of one of the alkynyl ligands, followed by nucleophilic attack of acetate to afford a vinyl ester is that of thermodynamic control.

Unexpectedly, the reaction of Os­(C≡C^t^Bu)_2_(=C=CH^t^Bu)­(P^i^Pr_3_)_2_ with 2-aminopyridine provides a new route for the selective synthesis of a 2,3-disubstitued imidazo­[1,2-*a*]­pyridine derivative starting from *tert*-butylacetylene and 2-aminopyridine. Current efforts are focused on understanding the reaction mechanism and exploring the scope of the transformation.

## Experimental Section

### General Information

All reactions were performed with rigorous exclusion of moisture and air, using an argon/vacuum manifold and standard Schlenk-tube or glovebox techniques. Complex OsH_6_(P^i^Pr_3_)_2_ (**1**) was prepared according to the published method.^19^ Instrumental methods, X-ray, theoretical calculations details, and NMR spectra (Figures S2–S28) are given in the Supporting Information. Coupling constants *J* and *N* are given in hertz.

#### Reaction of OsH_6_(P^i^Pr_3_)_2_ (**1**) with *tert*-Butylacetylene: Preparation of Os­(C≡C^t^Bu)_2_(=C=CH^t^Bu)­(P^i^Pr_3_)_2_ (**2**)

A solution of **1** (150 mg, 0.29 mmol) in toluene (10 mL) was treated with *tert*-butylacetylene (145 μL, 1.16 mmol). The resulting mixture was heated at 45 °C for 14 h. After this time, the solution was evaporated to dryness, yielding a purple oil. Cooling down (−20 °C) acetone solutions of the oil afforded the precipitation of dark purple crystals, which were dried under vacuum. Yield 170 mg (77%). Anal. Calcd. for C_36_H_70_OsP_2_ (%): C, 57.26; H, 9.34. Found: C, 57.35 H, 9.55. HRMS (electrospray, *m*/*z*) calc. for C_36_H_71_OsP_2_ [M + H]^+^: 757.4642; found: 757.4664. IR (cm^–1^): ν­(C=C) 1630 (s). ^1^H NMR (300.13 MHz, C_6_D_6_, 298 K): δ 3.32 (m, 6H, PCH), 1.44 (dvt, ^3^ *J*_H–H_ = 6.8, *N* = 13.2, 36H, PCH­(*C*H_3_)_2_), 1.33 (s, 18H, ^t^Bu), 1.19 (s, 9H, ^t^Bu), 0.61 (t, ^4^ *J*_H–P_ = 3.4, =CH). ^31^P­{^1^H} NMR (121.50 MHz, C_6_D_6_, 298 K): δ 14.7 (s). ^13^C­{^1^H} APT NMR (75.48 MHz, C_6_D_6_, 298 K): δ 268.0 (t, ^2^ *J*_C–P_ = 10.3, Os=C), 146.9 (br, Os-*C* *≡*C), 117.0 (t, ^4^ *J*_C–P_ = 4.6, Os=C=*C*H), 112.6 (s, Os–C*≡C*), 33.5, 32.3 (both s, CH_3_^t^Bu), 30.2, 27.5 (both s, C ^t^Bu), 24.5 (vt, *N* = 24.0, P*C*H), 20.6 (s, PCH­(*C*H_3_)_2_).

#### Dimerization of *tert*-Butylacetylene Catalyzed by **2**

In an argon-filled glovebox, an NMR tube was charged with **2** (6 mg, 0.008 mmol), *tert*-butylacetylene (40 μL, 0.32 mmol), mesitylene (internal standard) and toluene-*d*_8_ (0.3 mL). This tube was periodically checked by ^1^H NMR spectroscopy and after 3 days at 298 K the quantitative formation of (*Z*)-1,4-di-*t*-butylbutatriene is observed. Heating this tube for 5 h at 353 K (or performing the reaction at 323 K for 11 h) leads to the formation of a mixture of the cumulene and its (*Z*)-1,3-enyne isomer in a 30:70 ratio (or 70:30). ^1^H NMR data of (*Z*)-1,4-di-*t*-butylbutatriene (300 MHz, C_7_D_8_, 298 K): δ 5.45 (s, 2H, =CH), 1.07 (s, 18H, ^t^Bu). ^1^H NMR data of (*Z*)-^t^BuC≡CCH=CH^t^Bu (300 MHz, C_7_D_8_, 298 K): δ 5.54 (d, ^3^ *J*_H–H_ = 12.0, 1H, HC=), 5.40 (d, ^3^ *J*_H–H_ = 12.0, 1H, HC=), 1.22, 1.18 (both s, 9H each, ^t^Bu). Those data agree with those previously reported for these compounds.^12b^

#### Reaction of 2 with Trifluoromethanesulfonic Acid: Formation of [Os­(C≡C^t^Bu)_2_(≡CCH_2_^t^Bu)­(P^i^Pr_3_)_2_]­OTf (**3**)

A screw-cap NMR tube containing a solution of **2** (10 mg, 0.01 mmol) in dichloromethane-*d*_2_ (0.25 mL) and cooled at 233 K was treated with a solution of trifluoromethanesulfonic acid (1.2 μL, 0.01 mmol) in dichloromethane-*d*_2_ (0.15 mL). The NMR tube was immediately introduced into an NMR probe cooled at 233 K. The immediate and quantitative conversion of **2** to **3** was observed by ^1^H and ^31^P­{^1^H} NMR spectroscopy. HRMS (electrospray, *m*/*z*) calc. for C_36_H_71_OsP_2_ [M]^+^: 757.4642; found: 757.4624. ^1^H NMR (400.13 MHz, CD_2_Cl_2_, 233 K): δ 3.32 (m, 6H, PCH), 2.19 (br, 2H, CH_2_), 1.36 (dvt, ^3^ *J*_H–H_ = 7.3, *N* = 14.8, 36H, PCH­(*C*H_3_)_2_), 1.18 (s, 18H, ^t^Bu), 1.03 (s, 9H, ^t^Bu). ^31^P­{^1^H} NMR (161.98 MHz, CD_2_Cl_2_, 233 K): δ 47.0 (s). ^13^C­{^1^H} APT NMR (100.63 MHz, CD_2_Cl_2_, 233 K): δ 282.8 (s, Os*≡*C), 156.1 (s, Os-*C* *≡*C), 110.2 (t, ^3^ *J*_H–P_ = 8.1, Os–C*≡* *C*), 66.1 (s, Os*≡*C-*C*H_2_), 37.9, 34.5 (both s, C ^t^Bu), 30.7, 30.0 (both s, CH_3_^t^Bu), 25.2 (vt, *N* = 25.4, P*C*H), 20.2 (s, PCH­(*C*H_3_)_2_). ^19^F NMR (376.46 MHz, CD_2_Cl_2_, 233 K): δ −78.7 (s).

#### Reaction of **2** with DOTf

Two screw-cap NMR tubes were charged with **2** (10 mg, 0.01 mmol). To one of them was added dichloromethane-*d*_2_ (0.25 mL) and to the other dichloromethane (0.25 mL) and both were cooled to 233 K. At this temperature, solutions of trifluoromethanesulfonic acid-*d*_1_ (1.2 μL, 0.01 mmol) in dichloromethane-*d*_2_ (0.15 mL) and dichloromethane (0.15 mL), respectively, were added. The ^1^H and ^2^H NMR spectra showed the presence of **3-** ***d***_**1**_. The ^1^H NMR (400 MHz, CD_2_Cl_2_, 233 K) data were identical to those of complex **3** with the exception of the intensity of the signal at 2.18 ppm (1.2H, CH_2_). ^2^H NMR (61.42 MHz, CH_2_Cl_2_, 233 K): δ 2.16 (br, CDH).

#### Reaction of **2** with 3HF·NEt_3_: Preparation of OsF­(C≡C^t^Bu)_2_(≡CCH_2_^t^Bu)­(P^i^Pr_3_)_2_ (**4**)

Complex **2** (70 mg, 0.09 mmol) was dissolved in dichloromethane (5 mL). 3HF·NEt_3_ (5 μL, 0.03 mmol) was added and the resulting solution was stirred at room temperature for 5 min. Volatiles were removed under vacuum and the pale yellow solid obtained was dried under vacuum. Yield: 66 mg (94%). Yellow single crystals suitable for X-ray diffraction analysis were grown by cooling a solution of **4** in acetone at −20 °C. Anal. Calcd. for C_36_H_71_FOsP_2_ (%): C, 55.78; H, 9.23. Found: C, 55.55; H, 8.96. HRMS (electrospray, *m*/*z*) calc. for C_36_H_72_FOsP_2_ [M + H]^+^: 777.4703; found: 777.4686. IR (cm^–1^): ν­(C=C) 1636 (m). ^1^H NMR (300.13 MHz, C_6_D_6_, 298 K): δ 3.17 (m, 6H, PCH), 1.54 (dvt, ^3^ *J*_H–H_ = 6.9, *N* = 13.3, 36H, PCH­(*C*H_3_)_2_), 1.44 (br, 2H, OsCCH_2_), 1.42 (s, 18H, ^t^Bu), 0.98 (s, 9H, ^t^Bu). ^31^P­{^1^H} NMR (121.50 MHz, C_6_D_6_, 298 K): δ 14.6 (d, ^2^ *J*_P–F_ = 50.4). ^13^C­{^1^H} APT NMR (75.48 MHz, C_6_D_6_, 298 K): δ 283.6 (dt, ^2^ *J*_C–F_ = 127.7, ^2^ *J*_C–P_ = 10.2, Os*≡*C), 121.4 (br, Os-*C≡*C), 89.6 (dt, ^3^ *J*_C–F_ = 19.2, ^3^ *J*_C–P_ = 12.1, Os–C*≡* *C*), 65.7 (d, ^3^ *J*_C–F_ = 12.1, CH_2_), 33.0 (s, CH_3_^t^Bu), 32.4 (s, C ^t^Bu), 31.3 (s, CH_3_^t^Bu), 29.3 (s, C ^t^Bu), 25.4 (vt, *N* = 25.2, P*C*H), 20.1, 20.2 (both s, PCH­(*C*H_3_)_2_). ^19^F NMR (376.46 MHz, CD_2_Cl_2_, 298 K): δ −314.0 (t, ^2^ *J*_F–P_ = 46.0, Os–F).

#### Reaction of **2** with Acetic Acid: Preparation of Os­(C≡C^t^Bu)­{κ^2^-*O,C*-OC­(CH_3_)­OC=CH^t^Bu}­(=C=CH^t^Bu)­(P^i^Pr_3_)_2_ (**5**)

Acetic acid (7 μL, 0.12 mmol) was added to a solution of **2** (90 mg, 0.12 mmol) in pentane (5 mL). The solution color changes instantaneously from purple to pale brown. After 5 min of stirring at room temperature, the volatiles were removed, yielding a beige solid. Yield: 82 mg (84%). Yellow single crystals suitable for X-ray diffraction analysis were grown by cooling a solution of **4** in acetone at −20 °C. Anal. Calcd. for C_38_H_74_O_2_OsP_2_ (%): C, 55.99; H, 9.15. Found; C, 56.09; H, 9.10. HRMS (electrospray, *m*/*z*) calc. for C_38_H_73_O_2_OsP_2_ [M – H]^+^: 815.4697; found: 815.4726. IR (cm^–1^): ν­(C=C) 1634 (s). ^1^H NMR (300.13 MHz, C_6_D_6_, 298 K): δ 4.51 (br, 1H, Os–C­(O)=C*H*), 3.07 (br, 6H, PCH), 1.62 (s, 3H, CH_3_), 1.47 (dvt, ^3^ *J*_H–H_ = 6.7, *N* = 13.1, 18H, PCH­(*C*H_3_)_2_), 1.39 (s, 9H, ^t^Bu), 1.34 (s, 9H, ^t^Bu), 1.32 (dvt, ^3^ *J*_H–H_ = 6.3, *N* = 12.4, 18H, PCH­(*C*H_3_)_2_), 1.27 (s, 9H, ^t^Bu), 0.34 (t, ^4^ *J*_H–P_ = 3.1, 1H, =CH). ^31^P­{^1^H} NMR (121.50 MHz, C_6_D_6_, 298 K): δ −10.1 (s). ^13^C­{^1^H} APT NMR (75.48 MHz, C_6_D_6_, 298 K): δ 285.4 (t, ^2^ *J*_C–P_ = 11.2, Os=C), 176.9 (s, O_2_ *C*CH_3_), 172.6 (t, ^2^ *J*_C–P_ = 11.6, Os-*C*(O)=CH), 146.9 (br, Os-*C≡*C), 130.5 (br, Os–C­(O)=*C*H), 116.9 (t, ^3^ *J*_C–P_ = 3.5, Os=C=*C*H), 113.7 (s, Os–C*≡C*), 34.4 (s, C ^t^Bu), 33.5, 32.7, 32.3 (s, CH_3_^t^Bu), 30.2, 27.8 (both s, C ^t^Bu), 24.5 (vt, *N* = 23.6, P*C*H), 20.6, 20.3, 20.2 (all s, PCH­(*C*H_3_)_2_), 18.2 (s, O_2_C*C*H_3_).

#### Reaction of **2** with Acetic Acid-*d*_4_

A screw-cap NMR tube was charged with **2** (10 mg, 0.01 mmol) and benzene-*d*_6_ (0.4 mL). Acetic acid-*d*_4_ (0.6 μL, 0.01 mmol) was added. The ^1^H and ^31^P­{^1^H} NMR spectra shown the conversion of **2** to **5-** ***d***_**4**_. The ^1^H NMR (300.13 MHz, C_6_D_6_, 298 K) data were identical to those of complex **5** with the exception of the intensity of the signals at 4.51 ppm (0.4H, C­(O)=CH) and at 0.34 ppm (0.6H, =CH), and the absence of the resonance at 1.62 ppm corresponding to the methyl group of the acetate ligand. Same procedure, but using protic benzene was carried out in order to run the ^2^H NMR spectrum. ^2^H NMR (46.07 MHz, benzene, 298 K): δ 4.51 (br, CDH), 1.54 (br, O_2_CCD_3_), 0.42 (br, Os=C=CDMe).

#### Reaction of **2** with 2-Aminopyridine

2-aminopyridine (56 mg, 0.60 mmol) was added to a solution of **2** (150 mg, 0.20 mmol) in toluene (10 mL) and the resulting mixture was heated at 80 °C for 14 h. After that time, the solvent was removed under vacuum and the reaction crude was checked by ^1^H and ^31^P­{^1^H} NMR spectroscopies (Figures S25–S26). Subsequently the reaction crude was passed through a silica gel column and 2,3-dineopentylimidazo­[1,2-*a*]­pyridine (**6**) was eluted with gradients of pentane/diethyl ether. Solvent evaporation afforded **6** as an oil. Slow evaporation of a diethyl ether solution of **6** results in its crystallization.

#### Procedure for the Catalytic Formation of 2,3-Dineopentylimidazo­[1,2-*a*]­pyridine (**6**)

A screw-cap Schlenk flask was charged with 2-aminopyridine (150 mg, 1.60 mmol), *tert*-butylacetylene (200 μL, 1.60 mmol), complex **2** (30 mg, 0.04 mmol), and toluene (10 mL). The mixture was heated at 100 °C for 20 h and worked up following the procedure described above to afford 2,3-dineopentylimidazo­[1,2-*a*]­pyridine in 85% yield (170 mg). Anal. Calcd. for C_17_H_26_N_2_ (%): C, 79.02; H, 10.14; N, 10.84. Found; C, 78.98; H, 10.12; N, 10.80. HRMS (electrospray, *m*/*z*) calc. for C_17_H_27_N_2_ [M + H]^+^: 259.2169; found: 259.2169. ^1^H NMR (300.13 MHz, C_6_D_6_, 298 K): δ 7.55 (dt, ^3^ *J*_H–H_ = 9.0, ^4^ *J*_H–H_ = 1.2, 1H, CH_arom_), 7.46 (dt, ^3^ *J*_H–H_ = 6.9, ^4^ *J*_H–H_ = 1.2, 1H, CH_arom_), 6.60 (ddd, ^3^ *J*_H–H_ = 9.0, ^3^ *J*_H–H_ = 6.6, ^4^ *J*_H–H_ = 1.3, 1H, CH_arom_), 6.19 (td, ^3^ *J*_H–H_ = 6.9, ^4^ *J*_H–H_ = 1.3, 1H, CH_arom_), 2.86, 2.58 (both s, 2H each, CH_2_), 1.22, 0.79 (both s, 9H each, ^t^Bu). ^13^C­{^1^H} APT NMR (75.48 MHz, C_6_D_6_, 298 K): δ 145.0 (s, C), 144.6 (s, C), 123.7 (s, CH), 121.9 (s, CH), 119.8 (s, C), 117.8 (s, CH), 110.5 (s, CH), 42.0, 36.5 (both s, CH_2_), 35.2, 33.0 (both s, C ^t^Bu), 30.5, 30.1 (both s, CH_3_^t^Bu).

## Supplementary Material





## Notes

Cartesian coordinates of the optimized structures (XYZ)
General information for the Experimental Section, structural analysis, computational details, and NMR spectra (PDF)
