# Supplementary material for: Reactivity of a Bis(alkynyl)-Vinylidene Osmium Complex: Access to Carbynes, Vinyl Ester Derivatives, and C–C and C–N Bond-Forming Reactions
Source: Organometallics. 2026 Sep 22;45(19):2341–9. doi: 10.1021/acs.organomet.6c00277 (PMC13670176; doi:10.1021/acs.organomet.6c00277)
Supplement: Supplementary file 1 [file om6c00277_si_002.pdf]

SUPPORTING INFORMATION

Reactivity of a Bis(alkynyl)-Vinylidene Osmium Complex: Access to Carbynes, Vinyl Ester Derivatives, and C–C and C–N Bond-Forming Reactions

Sheila G. Curto,* Joaquín M. Granados, Enrique Oñate, and Montserrat Oliván*

Departamento de Química Inorgánica - Instituto de Síntesis Química y Catálisis Homogénea (ISQCH) - Centro de Innovación en Química Avanzada (ORFEO-CINQA), Universidad de Zaragoza - CSIC, 50009 Zaragoza, Spain

* e-mail: sheilagc@unizar.es (S.G.C.), molivan@unizar.es (M.O.)

Contents:

- Experimental Section: General Information	S2
- Structural Analysis of Complexes **2**, **3**, **4**, **5**, and **6**	S3
- Computational Details	S5
- NMR spectra	S7
- References	S21

• Experimental Section: General Information

All reactions were performed with rigorous exclusion of air using standard Schlenk-tube or glovebox techniques. Solvents were dried by the usual procedures and distilled under argon before use or obtained oxygen- and water-free from an MBraun solvent purification apparatus. Toluene-d_8 and benzene-d_6 were stored over sodium, and dichloromethane-d_2 over molecular sieves. Complex $OsH_6(P^iPr_3)_2$ was prepared according to the published method.[1] *tert*-Butylacetylene and 2-aminopyridine were purchased from commercial sources and flash distilled and recrystallized, respectively, prior to use. C, H, and N analyses were carried out in a PerkinElmer 2400 CHNS/O analyzer. High-resolution (HRMS) electrospray mass spectra were acquired using a MicroTOF-Q hybrid quadrupole time-of-flight spectrometer (Bruker Daltonics, Bremen, Germany). Attenuated total reflection infrared spectra (ATR-IR) of solid samples were run on a Perkin-Elmer Spectrum 100 FT-IR spectrometer. ^{1}H, ^{2}H, ^{13}C{^{1}H}, ^{31}P{^{1}H}, and ^{19}F{^{1}H} NMR spectra were recorded on Bruker 300 ARX, Bruker Avance 300 MHz or Bruker Avance 400 MHz instruments. Chemical shifts (expressed in ppm) are referenced to residual solvent peaks (^{1}H, ^{2}H, ^{13}C{^{1}H}), external 85% H_3PO_4 (^{31}P{^{1}H}), or external $CFCl_3$ (^{19}F{^{1}H} NMR). Coupling constants J and N ($N = J_{P\text{-}H} + J_{P'\text{-}H}$ for ^{1}H and $N = J_{P\text{-}C} + J_{P'\text{-}C}$ for ^{13}C{^{1}H}) are given in hertz.

• Structural Analysis of Complexes 2, 3, 4, 5, and 6

X-ray data were collected on a D8 Venture Bruker diffractometers (Mo radiation, λ = 0.71073 Å). The crystals were mounted under oil in a MiTeGen mount and cooled to 100(2) K (or 162(2) K (**2**)) with an open-flow nitrogen gas (Oxford Cryosystems). Data were corrected for absorption by using a multiscan method applied with the SADABS program.[2] The structures were solved by direct methods and refined by full-matrix least squares on F^2 with SHELXL2019,[3] including isotropic and subsequently anisotropic displacement parameters. The hydrogen atoms were observed in the last Fourier Maps or calculated, and refined freely or using a restricted riding model.

The disordered groups of **2**, **3**, and **4** were refined with different moieties, restricted geometry and restricted isotropic or anisotropic thermal parameters.

The crystals of **4** were observed twined. The cell for domain 2 is rotated from domain 1 by 179.9 degrees about reciprocal axis 1.000 -0.002 0.002 and real axis 1.000 0.040 0.007. The molecule was refined with HKLF5 with about 0.57/0.43 ratio for the two components.

Crystal data for **2** (CCDC 2577024): $C_{36}H_{70}OsP_2$, M_W 755.06, black, irregular block (0.196 x 0.133 x 0.117 mm^3), monoclinic, space group Cc, *a*: 12.4578(9) Å, *b*: 19.9641(12) Å, *c*: 16.5733(11) Å, β: 109.748(2)°, V = 3879.5(4) $Å^3$, Z = 4, Z' = 1, D_{calc}: 1.293 g cm^{-3}, F(000): 1568, T = 162(2) K, μ 3.390 mm^{-1}. 49250 measured reflections (2θ: 3-57°, ω and φ scans 0.5°), 9078 unique (R_{int} = 0.0345); min./max. transm. factors 0.617/0.746. Final agreement factors were R^1 = 0.0488 (8542 observed reflections, I > 2σ(I)) and wR^2 = 0.1224; Flack parameter 0.05(2); data/restraints/parameters 9078/43/353; GoF = 1.110. Largest peak and hole 2.466 (close to Os atoms) and -1.606 e/ $Å^3$.

Crystal data for **3** (CCDC 2577027): $C_{37}H_{71}F_3O_3OsP_2S$, M_W 905.13, blue, irregular block, (0.117 x 0.098 x 0.095 mm^3), monoclinic, space group $P2_1/n$, *a*: 12.6568(5) Å, *b*: 22.8714(8) Å, *c*: 15.5768(6) Å, β: 94.3131(15)°, V = 4496.4(3) $Å^3$, Z = 4, Z' = 1, D_{calc}: 1.337 g cm^{-3}, F(000): 1864, T = 100(2) K, μ 2.996 mm^{-1}. 175210 measured reflections (2θ: 3-57°, ω and φ scans 0.5°), 11163 unique (R_{int} = 0.0476); min./max. transm. factors 0.665/0.746. Final agreement factors were R^1 = 0.0371 (10372 observed reflections, I > 2σ(I)) and wR^2 = 0.0911; data/restraints/parameters 11163/24/433; GoF = 1.067. Largest peak and hole 3.664 (close to Os atoms) and -2.225 e/ $Å^3$.

Crystal data for **4** (CCDC 2577025): $C_{36}H_{71}FOsP_2$, M_W 775.06, yellow, irregular block, (0.154 x 0.122 x 0.037 mm^3), triclinic, space group P-1, *a*: 12.7154(7) Å, *b*: 16.4602(10) Å, *c*: 18.6675(11) Å, *a*: 94.321(2)°, *β*: 90.238(2)°,*γ*: 93.065(3)°, *V* = 3890.3(4) $Å^3$, *Z* = 4, *Z'* = 2, D_{calc}: 1.323 g cm^{-3}, F(000): 1608, T = 100(2) K, μ 3.386 mm^{-1}. 151093 measured reflections (2θ: 3-57º, ω and φ scans 0.5º), 151093 unique (R_{int} = 0.0377); min./max. transm. factors 0.615/0.746. Final agreement factors were R^1 = 0.0373 (19733 observed reflections, I > 2σ(I)) and wR^2 = 0.0965; data/restraints/parameters 21645/72/794; GoF = 1.115. Largest peak and hole 4.144 (close to Os atoms) and -1.877 e/ $Å^3$.

Crystal data for **5** (CCDC 2577028): $C_{38}H_{74}O_2OsP_2$, M_W 815.11, yellow, irregular block, (0.194 x 0.157 x 0.062 mm^3), monoclinic, space group $P2_1/c$, *a*: 22.2090(7) Å, *b*: 17.2364(5) Å, *c*: 22.3037(6) Å, *β*: 103.1475(11)°, *V* = 8314.1(4) $Å^3$, *Z* = 8, *Z'* = 2, D_{calc}: 1.302 g cm^{-3}, F(000): 3392, T = 100(2) K, μ 3.172 mm^{-1}. 284227 measured reflections (2θ: 3-57º, ω and φ scans 0.5º), 20651 unique (R_{int} = 0.0493); min./max. transm. factors 0.635/0.746. Final agreement factors were R^1 = 0.0262 (18053 observed reflections, I > 2σ(I)) and wR^2 = 0.0712; data/restraints/parameters 20651/0/ 819; GoF = 0.896. Largest peak and hole 2.196 (close to Os atoms) and -1.015 e/ $Å^3$.

Crystal data for **6** (CCDC 2577026): $C_{17}H_{26}N_2$, M_W 258.40, colorless, irregular block, (0.262 x 0.162 x 0.100 mm^3), monoclinic, space group $P2_1/c$, *a*: 14.4664(9) Å, *b*: 9.1211(5) Å, *c*: 12.1572(7) Å, *β*: 103.393(2)°, *V* = 1560.51(16) $Å^3$, *Z* = 4, *Z'* = 1, D_{calc}: 1.100 g cm^{-3}, F(000): 568, T = 100(2) K, μ 3.172 mm^{-1}. 39440 measured reflections (2θ: 3-57º, ω and φ scans 0.5º), 3879 unique (R_{int} = 0.0456); min./max. transm. factors 0.631/0.746. Final agreement factors were R^1 = 0.0420 (3341 observed reflections, I > 2σ(I)) and wR^2 = 0.1164; data/restraints/parameters 3879/0/178; GoF = 1.069. Largest peak and hole 0.319 and -0.224 e/ $Å^3$.

• Computational Details

All mechanistic calculations were performed at the DFT level using the B3LYP functional[4] supplemented with the Grimme's dispersion correction D3[5] as implemented in Gaussian09.[6] Os atoms were described by means of an effective core potential SDD for the inner electron[7] and its associated double-ζ basis set for the outer ones, complemented with a set of f-polarization functions for osmium.[8] The 6-31G** basis set was used for all the other atoms.[9] The SMD continuum model was used to model the effects of the solvent. The solvents used were CH_2Cl_2 and *n*-pentane. The coordinates included in the xyz file correspond to those calculated in CH_2Cl_2. Reactants, intermediates, and products were also characterized by frequency calculations and has positive definite Hessian matrices thus confirming that the computed structure is a minimum on the potential energy surface. Transition states were identified by having one imaginary frequency in the Hessian matrix. It was confirmed that transition states connect with the corresponding intermediates by means of application of an eigenvector corresponding to the imaginary frequency and subsequent optimization of the resulting structures. Gibbs energies were computed at 298.15 K and 1 atmosphere. All values collected in figures correspond to Gibbs energies in kcal mol^{-1}. The NBO calculations were performed by the NBO7 program.[10]

Table S1. Energies of Optimized Molecules

**Molecule**	**Energy: sum of electronic and thermal Free Energies (Hartree/Molecule)**	
Solvent (scrf)	CH_2Cl_2	*n*-pentane
**2**	-2186.77668	-2186.763515
**TS$_{2-A}$**	-2415.827364	-2415.816258
**A**	-2415.834358	-2415.821986
**TS$_{A-B}$**	-2415.802785	-2415.794895
**B**	-2415.808546	-2415.798138
**TS$_{B-C}$**	-2415.802702	-2415.793437
**C**	-2415.821504	-2415.811051
**TS$_{C-5}$**	-2415.818199	-2415.809656
**5**	-2415.848995	-2415.839967
**TS$_{2-3Ac}$**	-2415.819193	-2415.801404
**3$_{Ac}$**	-2415.818377	-2415.798144
**4$_{Ac}$**	-2415.826071	-2415.812566
**CH_3COOH**	-229.063535	-229.060291
**3$_F$**	-2287.180927	
**4**	-2287.232172	
**HF**	-100.440366	

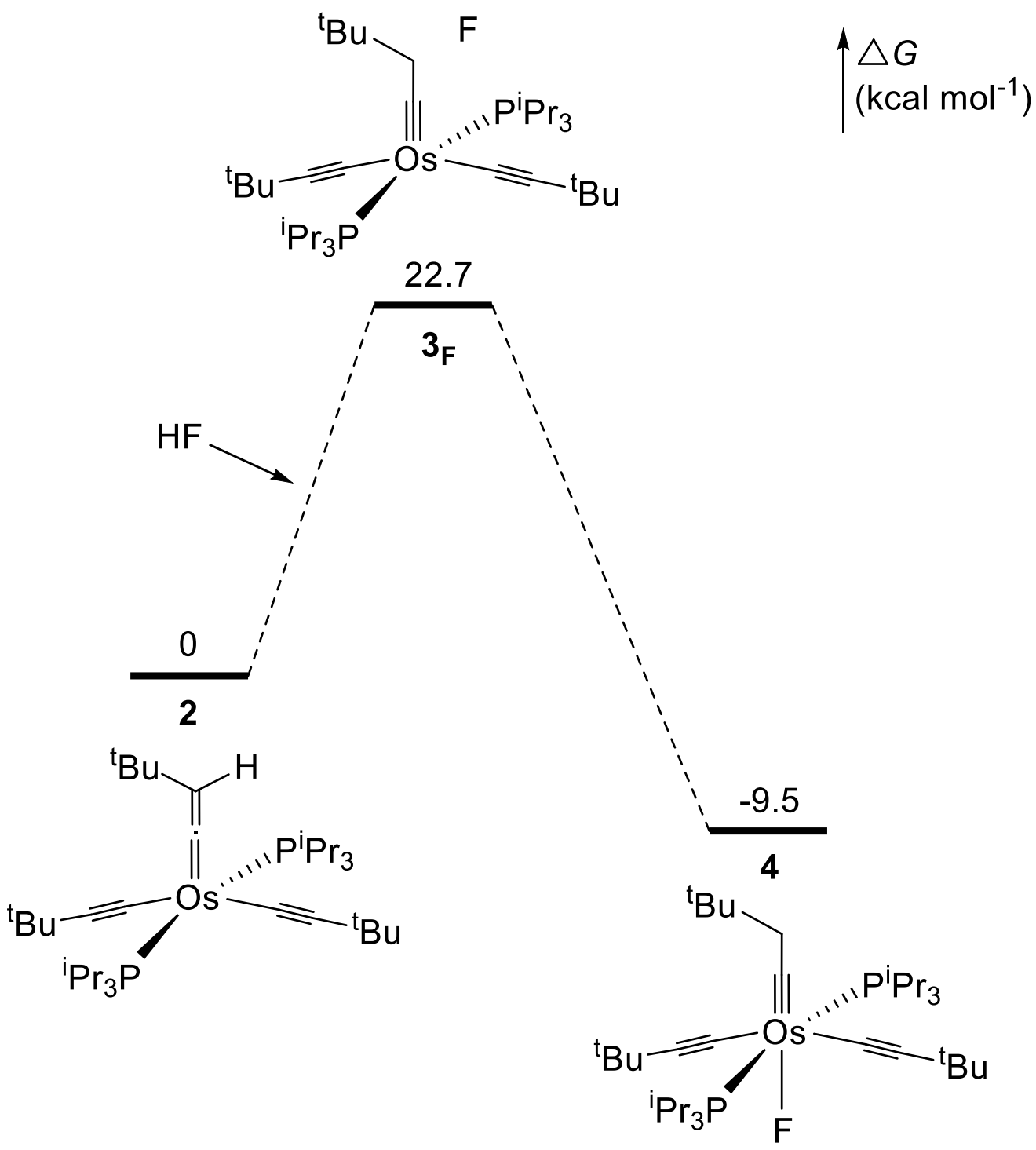

Figure S1. Computed energy profile (ΔG in kcal mol^{-1}) for the reaction of **2** with HF in dichloromethane.

• NMR spectra

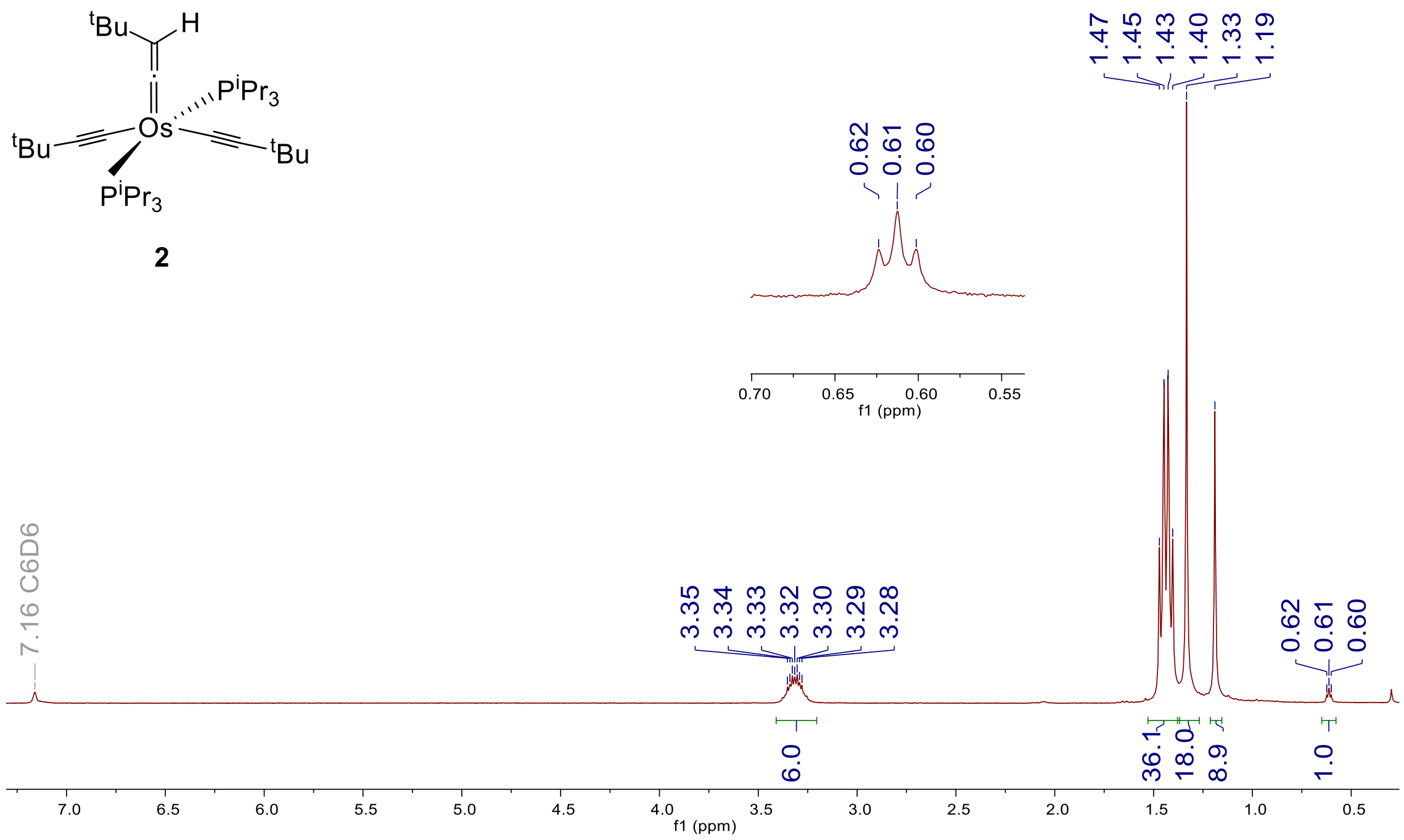

Figure S2. ^{1}H NMR spectrum (300.13 MHz, C_6D_6, 298 K) of complex **2**.

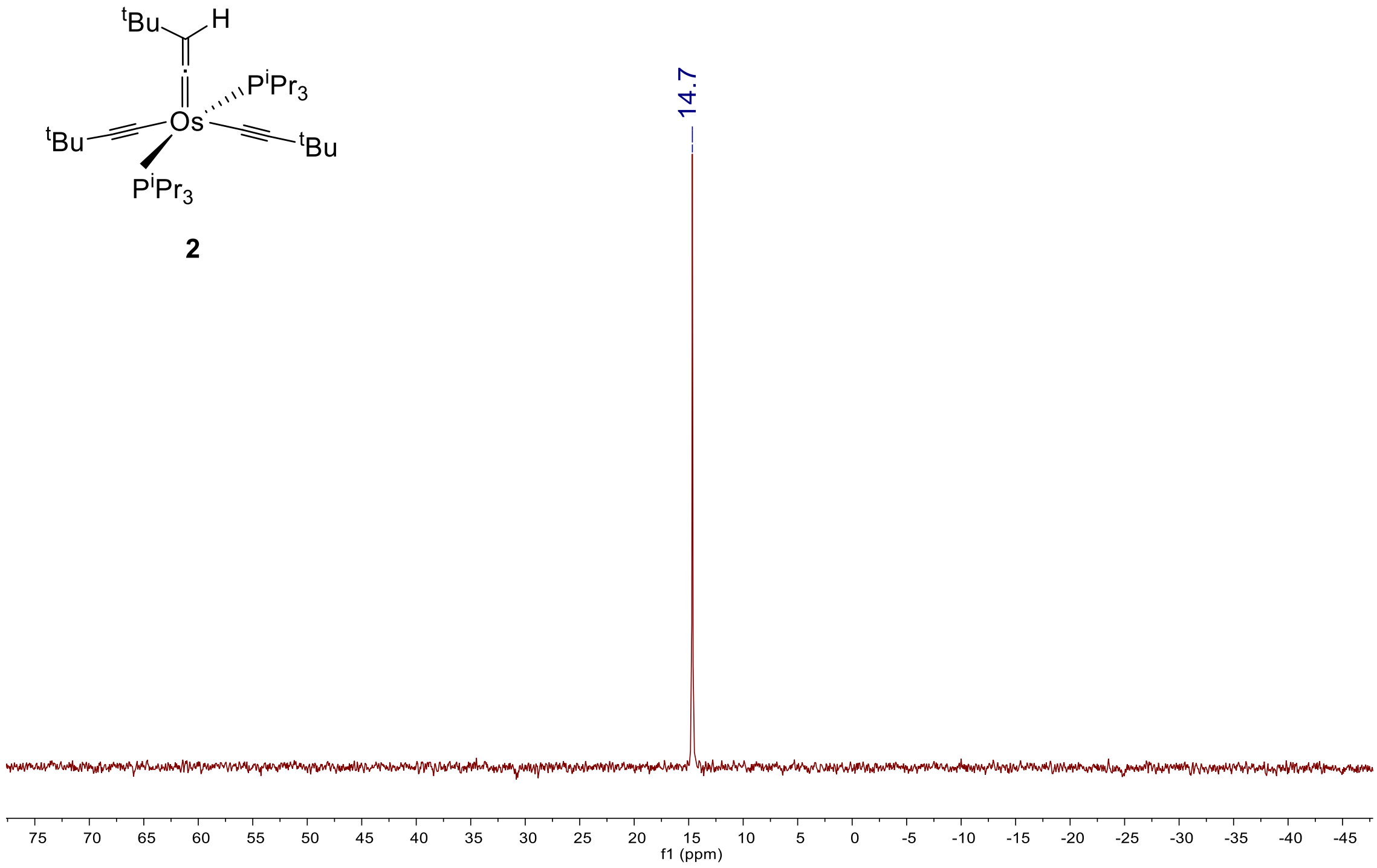

Figure S3. $^{31}P\{^{1}H\}$ NMR spectrum (121.50 MHz, C_6D_6, 298 K) of complex **2**.

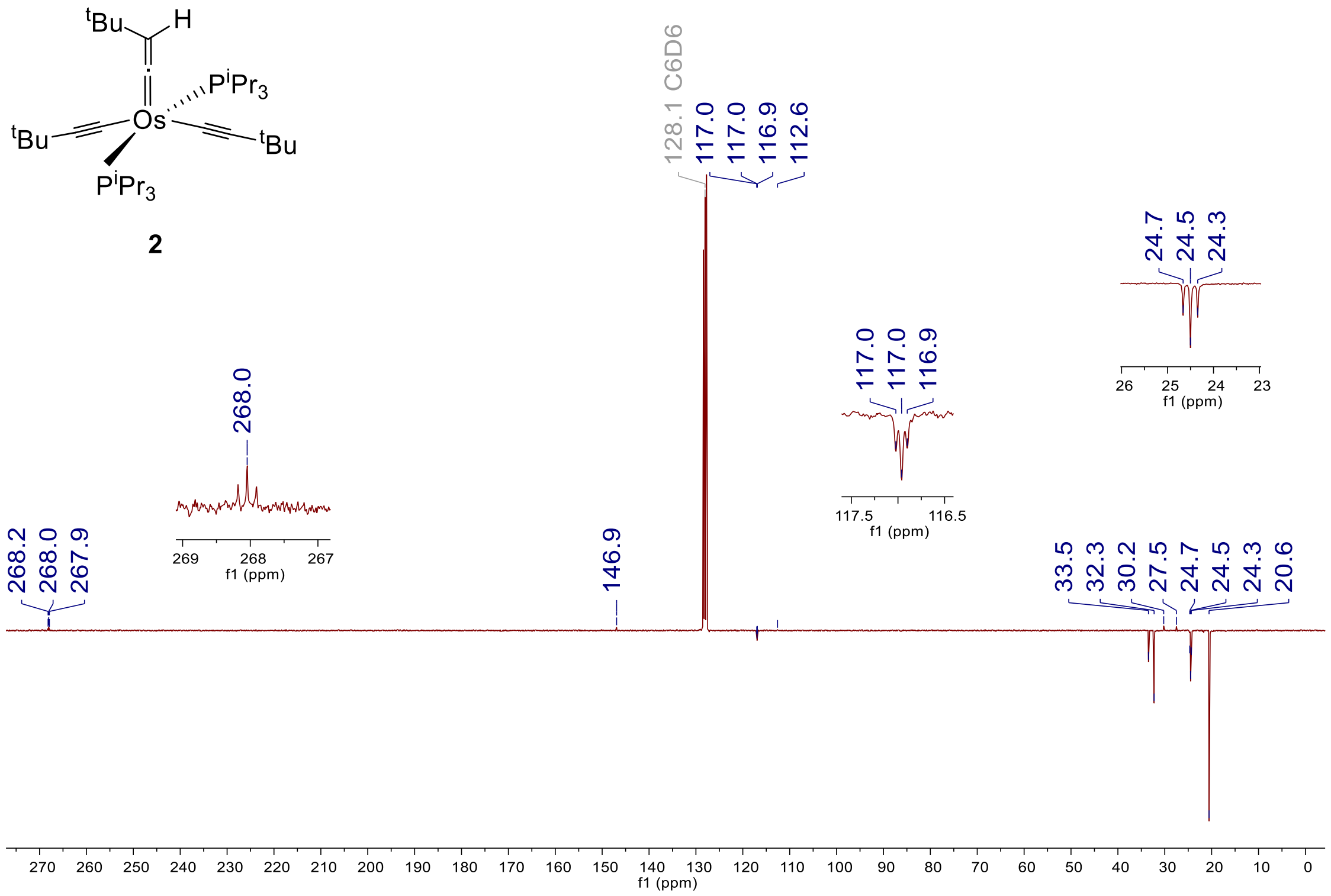

Figure S4. $^{13}C\{^{1}H\}$ APT NMR spectrum (75.48 MHz, C_6D_6, 298 K) of complex **2**.

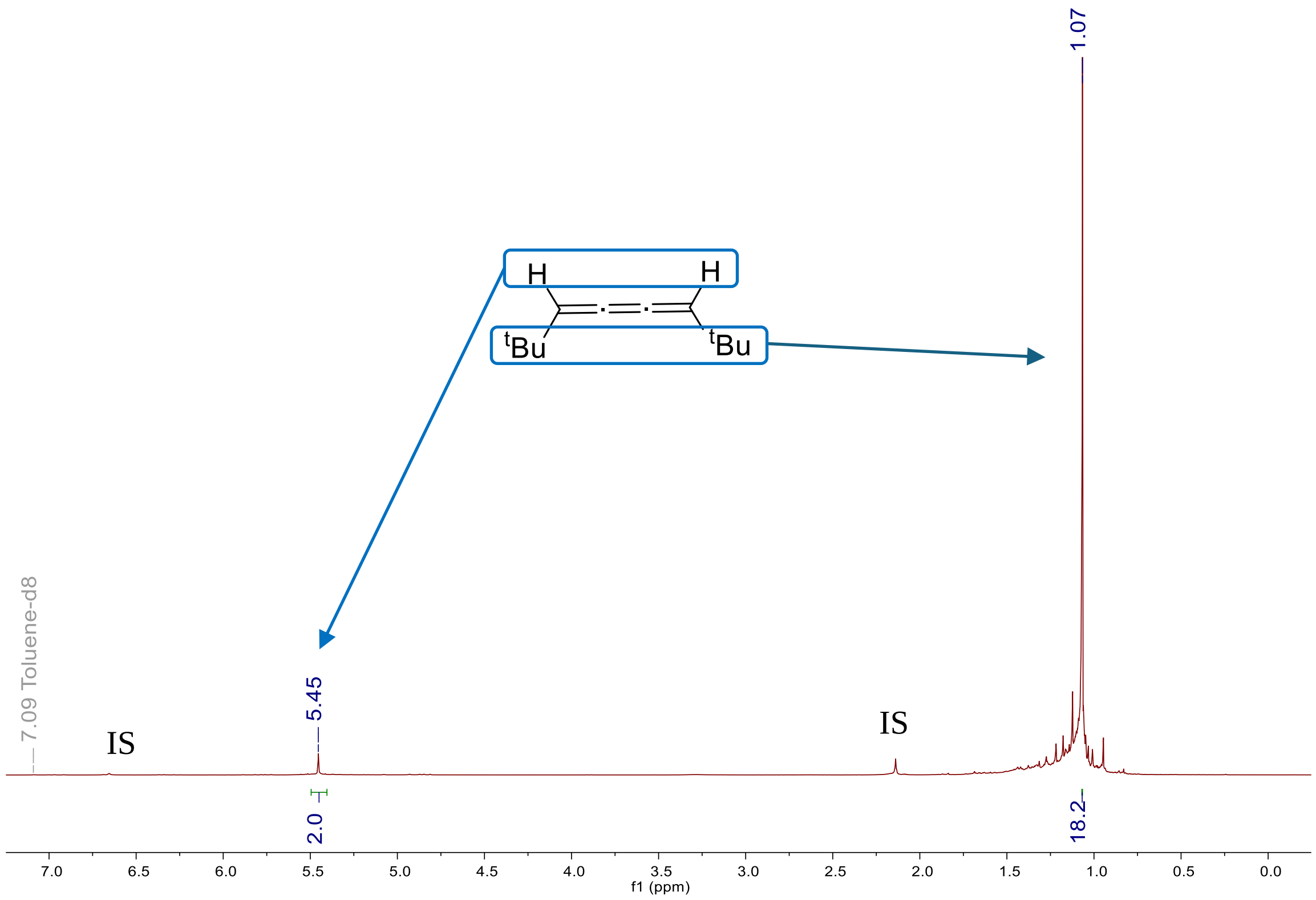

Figure S5. ^{1}H NMR spectrum (300.13 MHz, C_7D_8, 298 K) of the crude of the dimerization of *t*-butylacetylene catalyzed by **2**, after 3 days at 298 K (mesitylene used as internal standard).

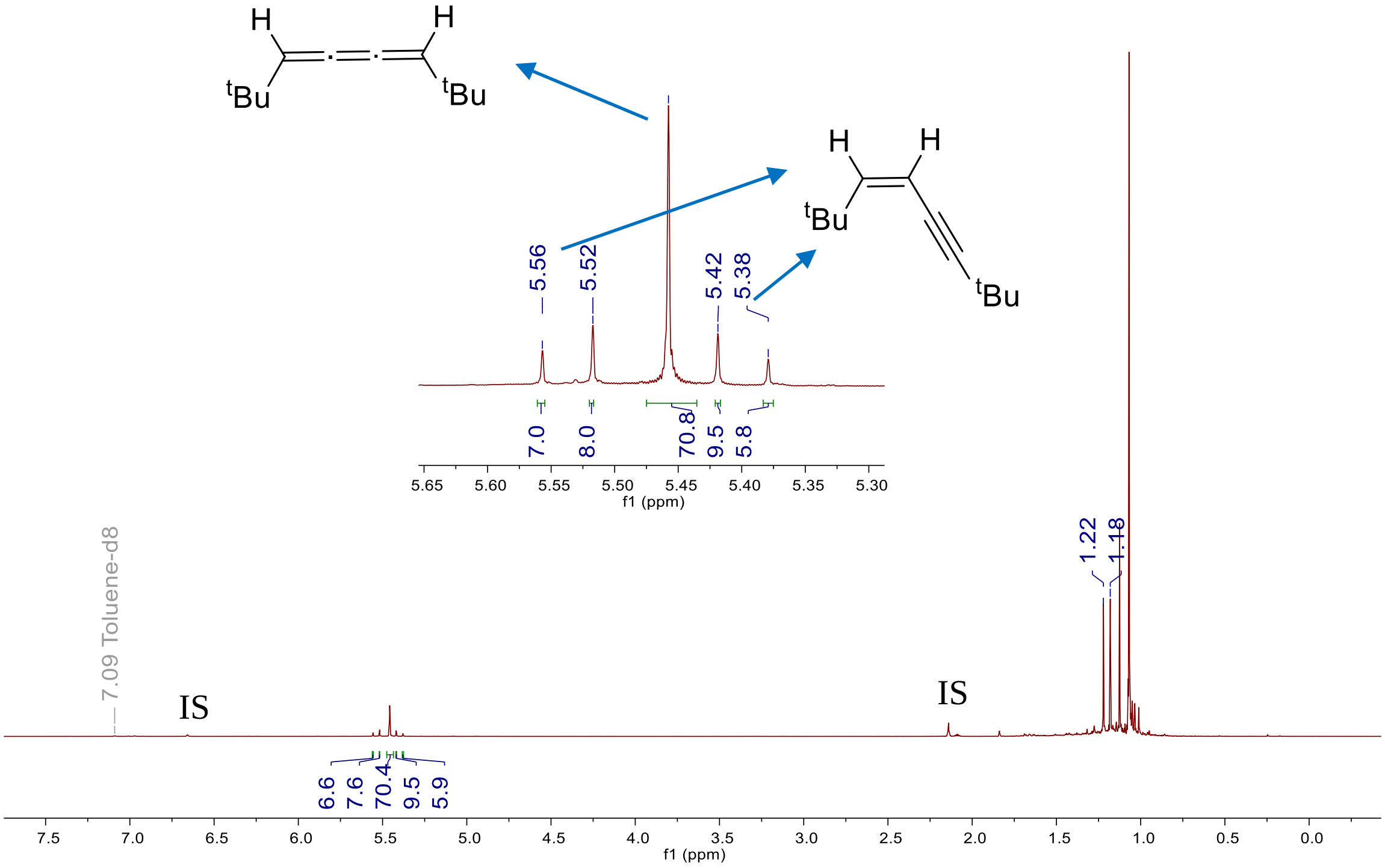

Figure S6. ^{1}H NMR spectrum (300.13 MHz, C_7D_8, 298 K) of the crude of the dimerization of *t*-butylacetylene catalyzed by **2**, after 11 h at 323 K (mesitylene used as internal standard).

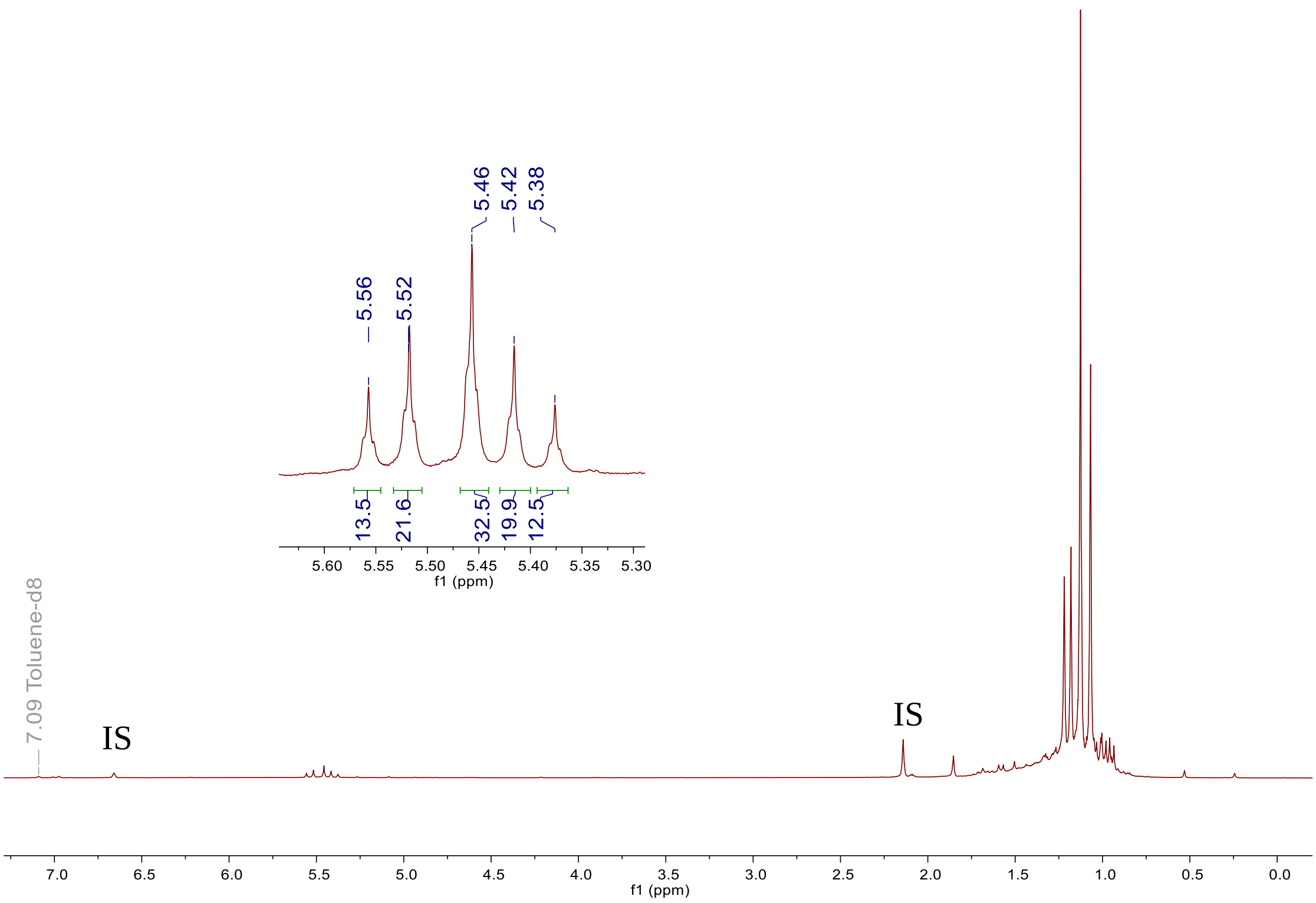

Figure S7. ^{1}H NMR spectrum (300.13 MHz, C_7D_8, 298 K) of the crude of the dimerization of *t*-butylacetylene catalyzed by **2**, after 3 days at 298 K and 5 additional hours at 353 K (mesitylene used as internal standard).

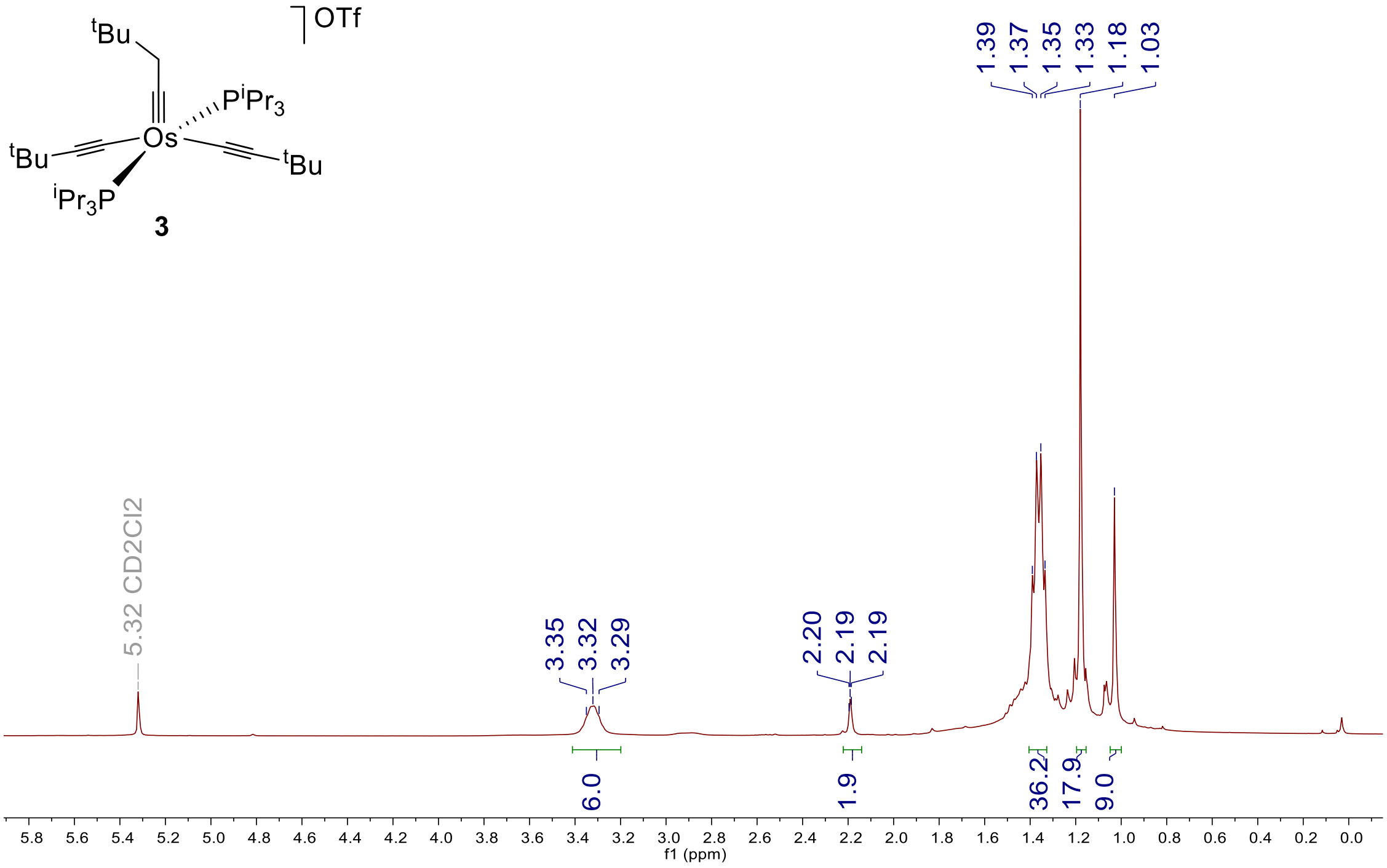

Figure S8. ^{1}H NMR spectrum (400.13 MHz, CD_2Cl_2, 233 K) of complex **3**.

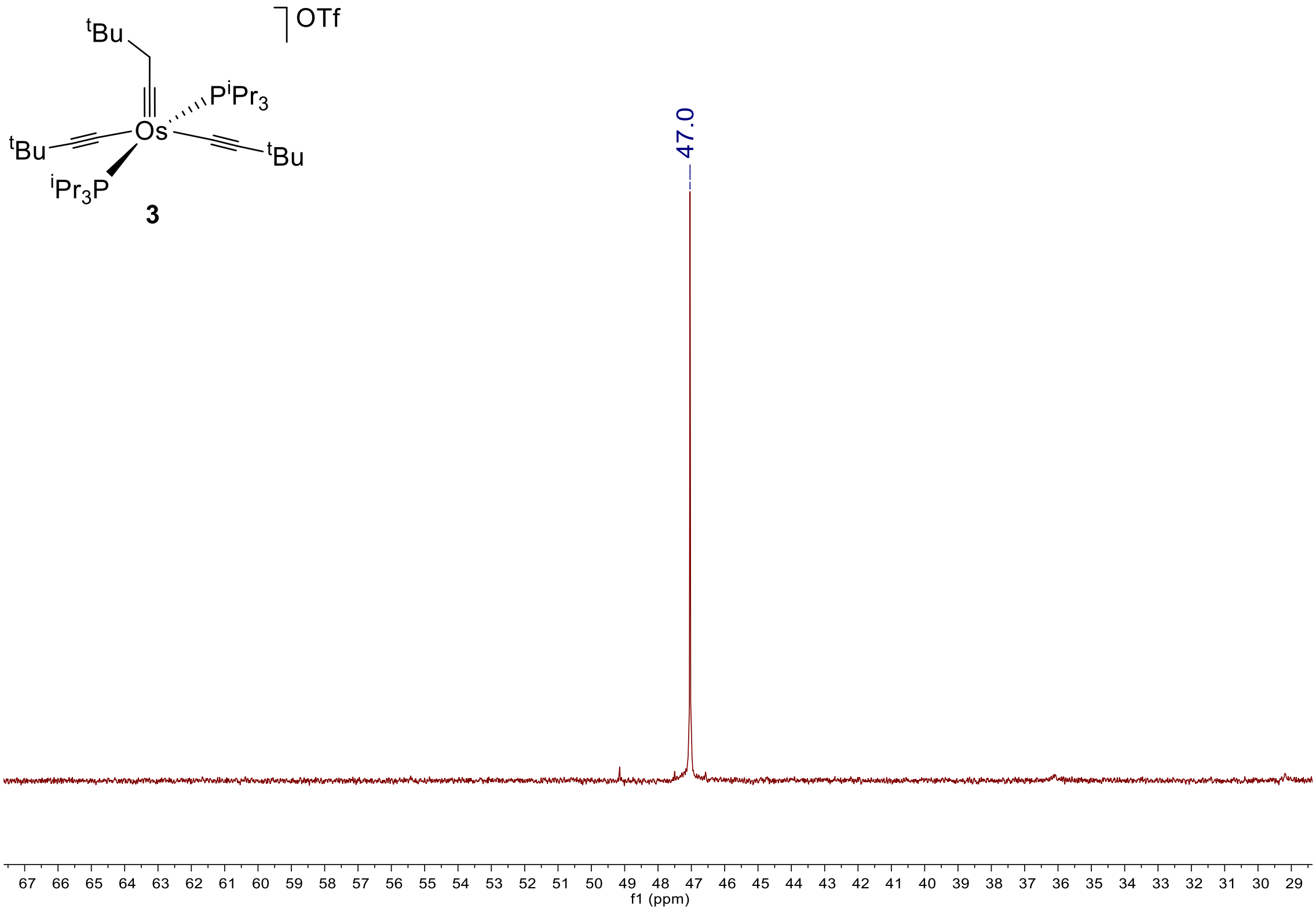

Figure S9. ^{31}P{^{1}H} NMR spectrum (161.98 MHz, CD_2Cl_2, 233 K) of complex **3**.

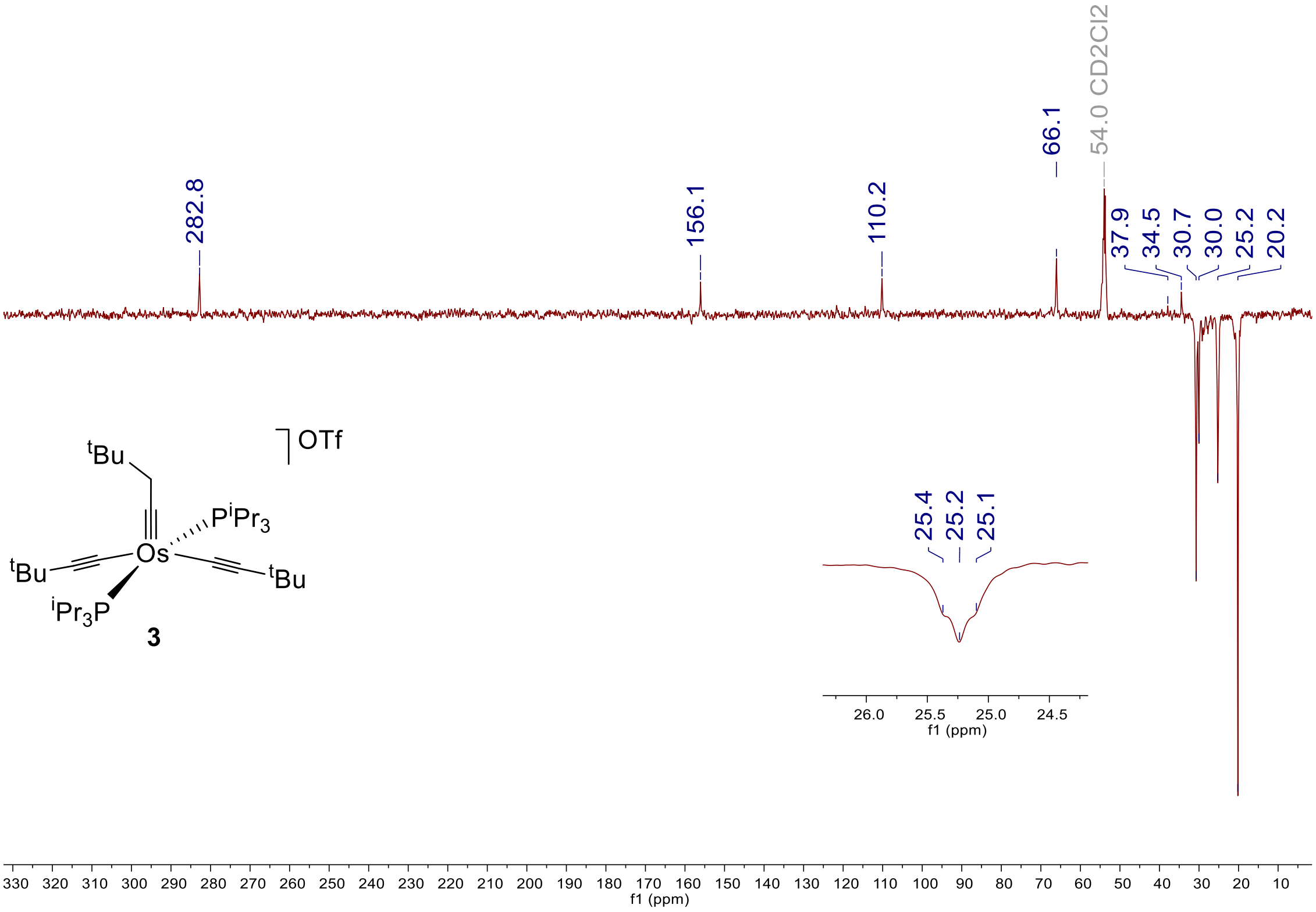

Figure S10. $^{13}C\{^{1}H\}$ APT NMR spectrum (100.63 MHz, CD_2Cl_2, 233 K) of complex **3**.

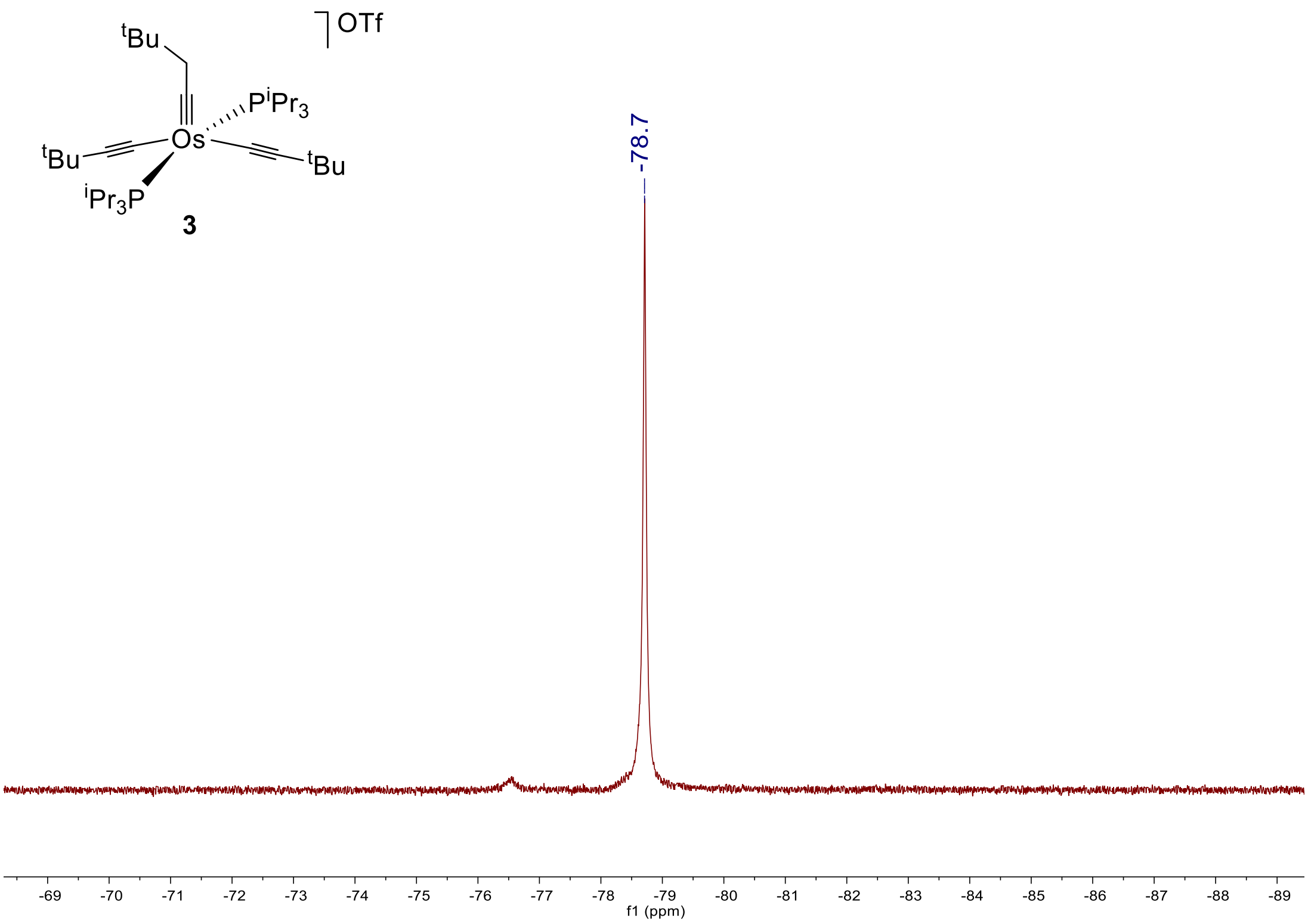

Figure S11. ^{19}F NMR spectrum (376.46 MHz, CD_2Cl_2, 233 K) of complex **3**.

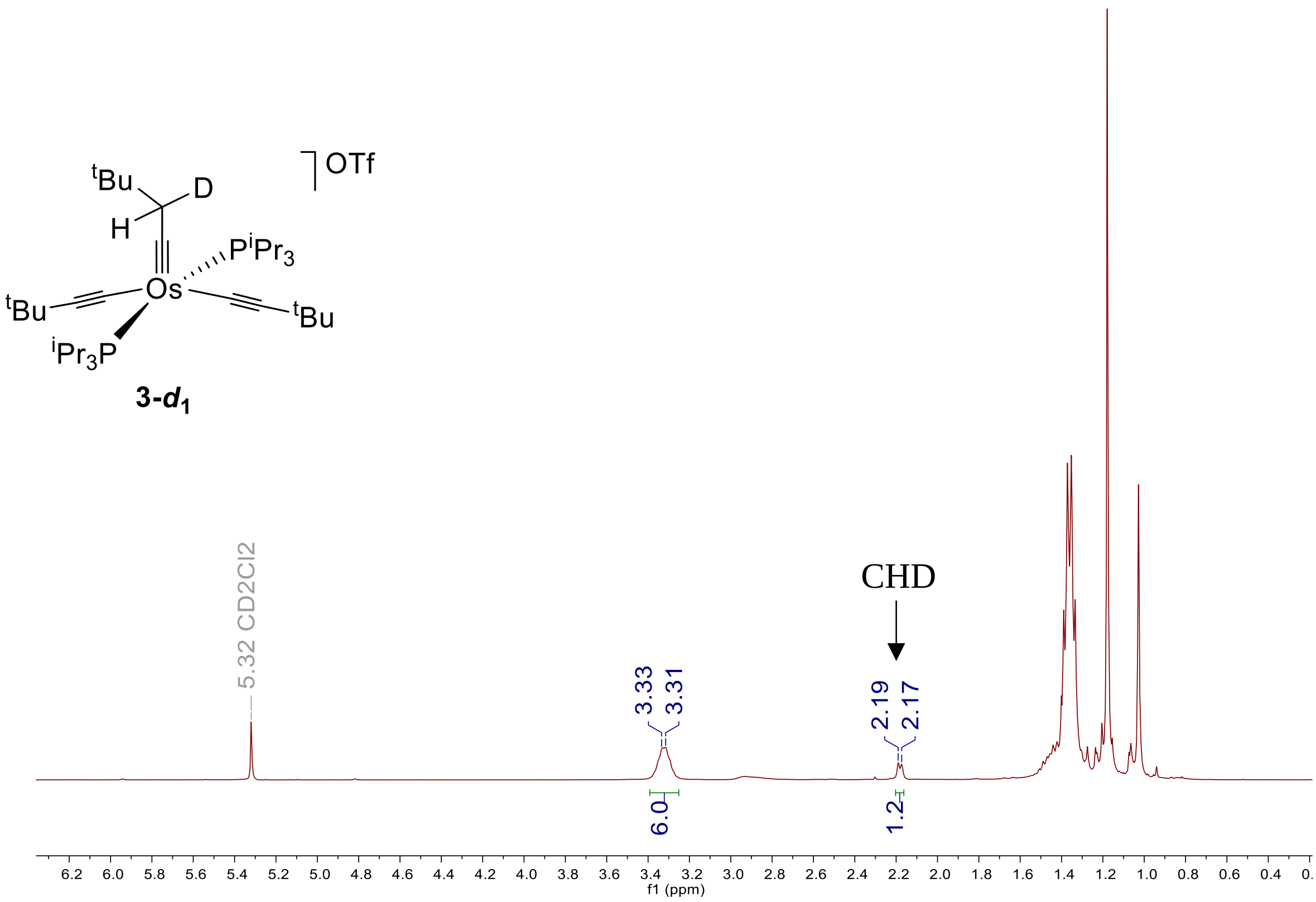

Figure S12. ¹H NMR spectrum (400.13 MHz, CD_2Cl_2, 233 K) of complex **3-*d*$_1$**.

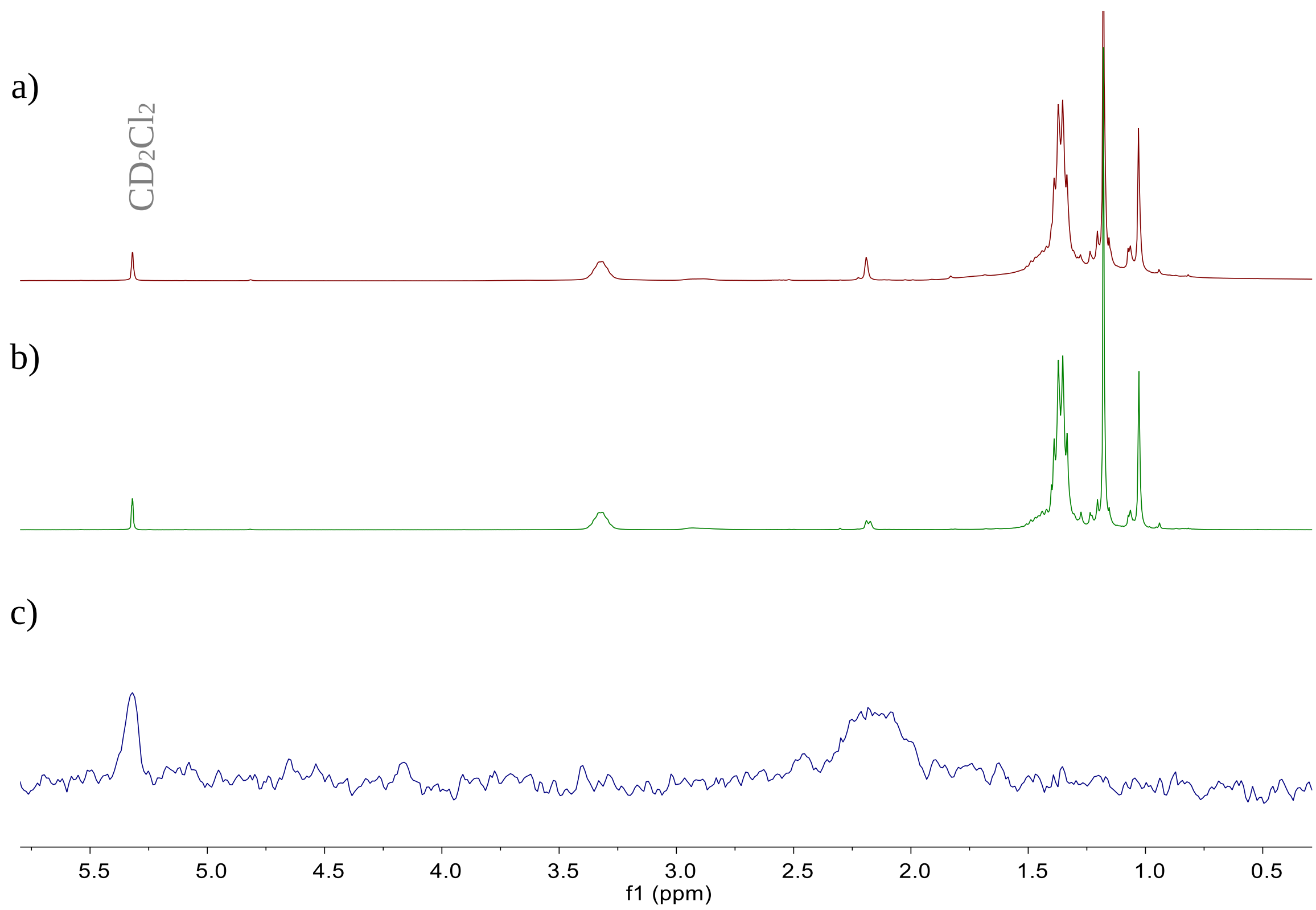

Figure S13. a) ¹H NMR spectrum (400.13 MHz, CD_2Cl_2, 233 K) of complex **3**; b) ¹H NMR spectrum (400.13 MHz, CD_2Cl_2, 233 K) of complex **3-*d*$_1$**; c) ²H NMR spectrum (61.42 MHz, CH_2Cl_2, 233 K) of complex **3-*d*$_1$**.

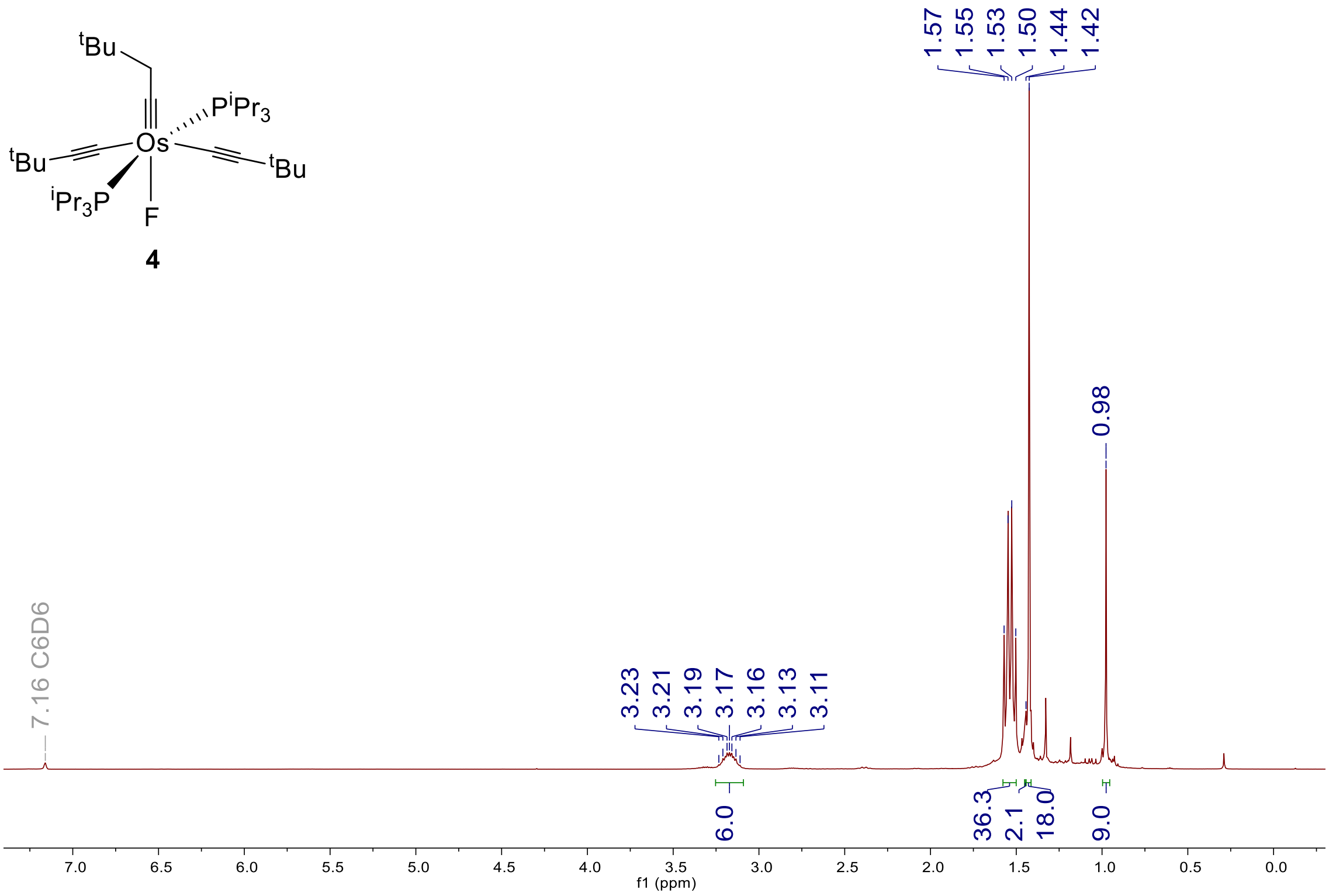

Figure S14. ^{1}H NMR spectrum (300.13 MHz, C_6D_6, 298 K) of complex **4**.

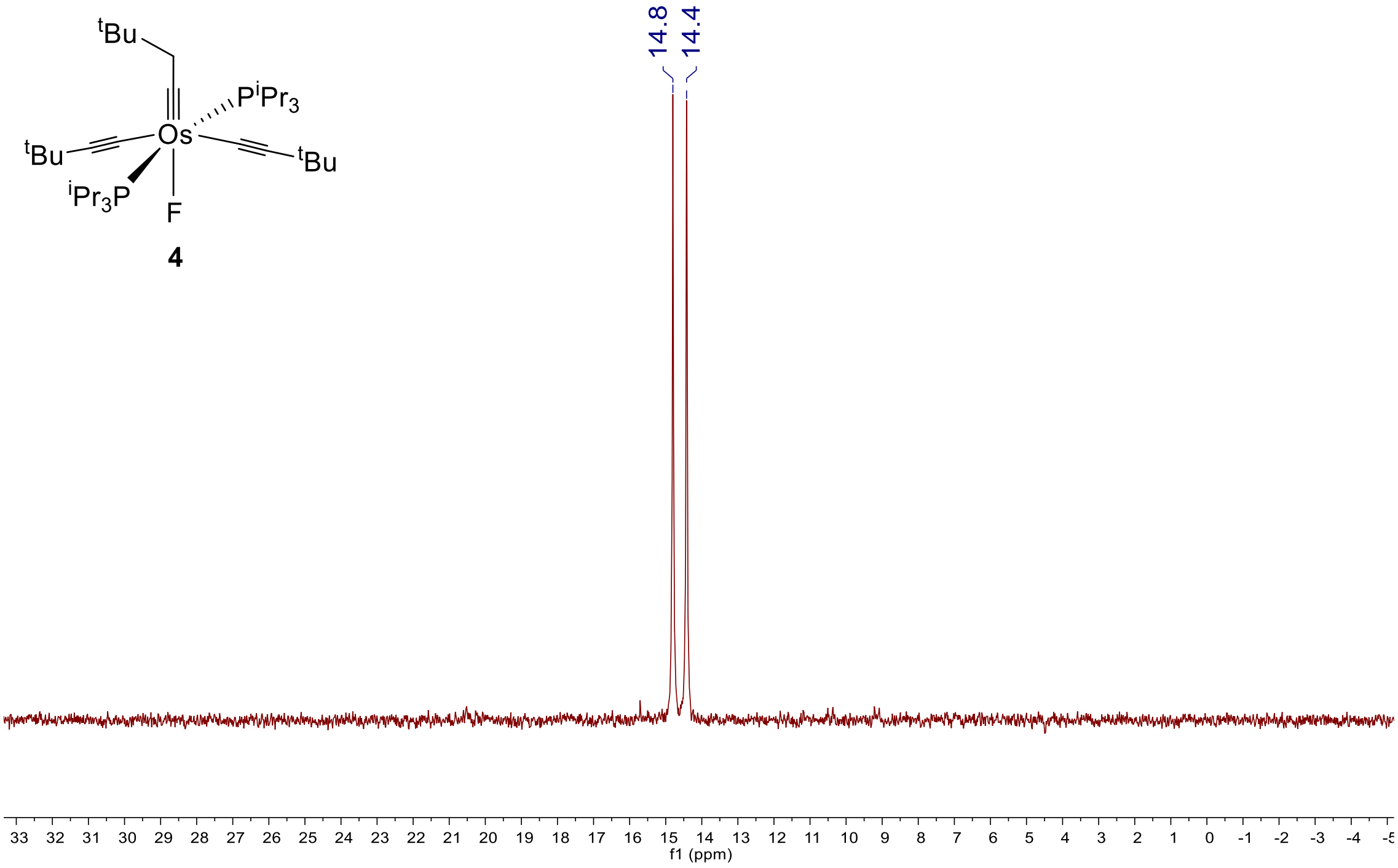

Figure S15. $^{31}P\{^{1}H\}$ NMR spectrum (121.50 MHz, C_6D_6, 298 K) of complex **4**.

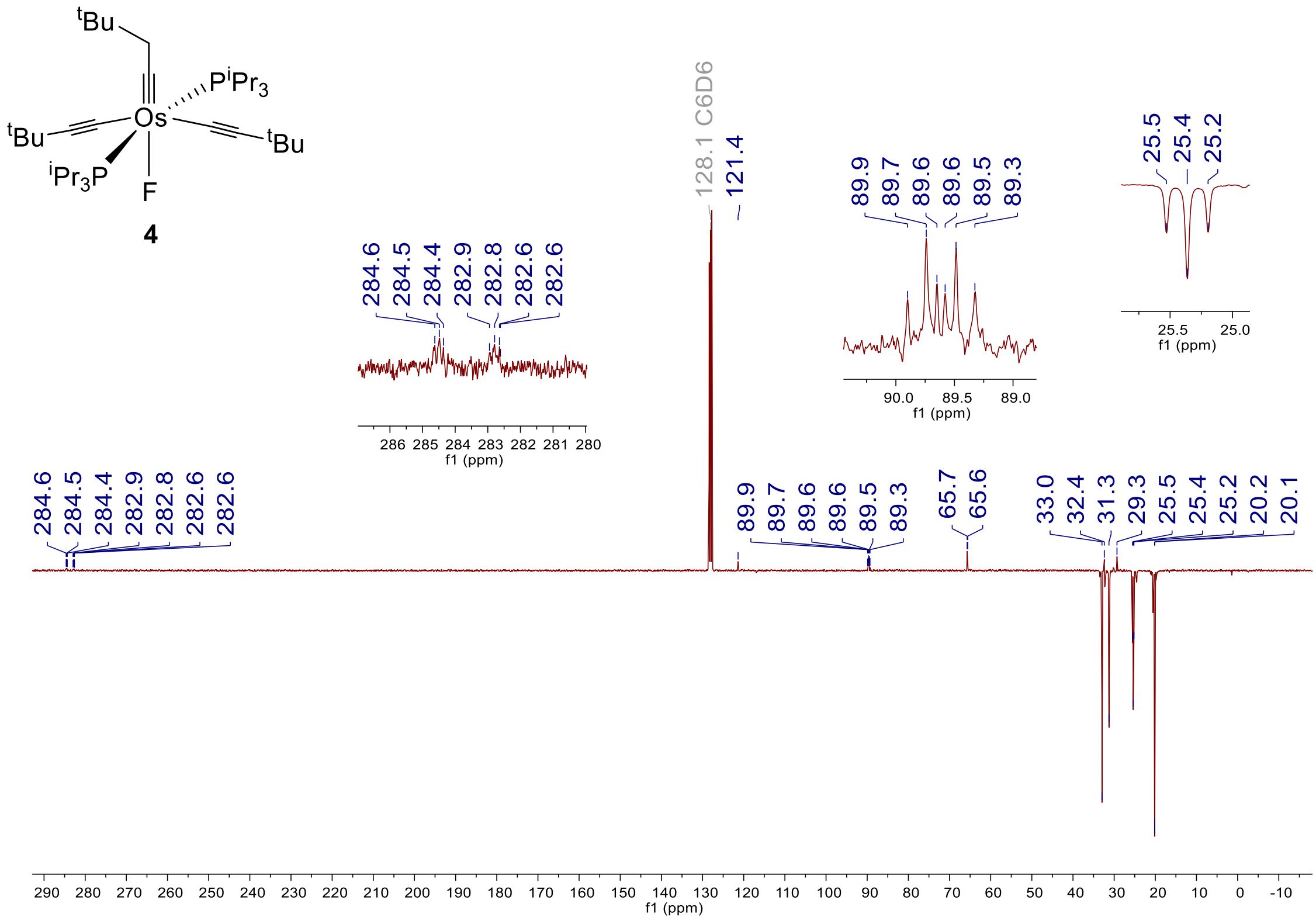

Figure S16. $^{13}C\{^{1}H\}$ APT NMR spectrum (75.48 MHz, C_6D_6, 298 K) of complex **4**.

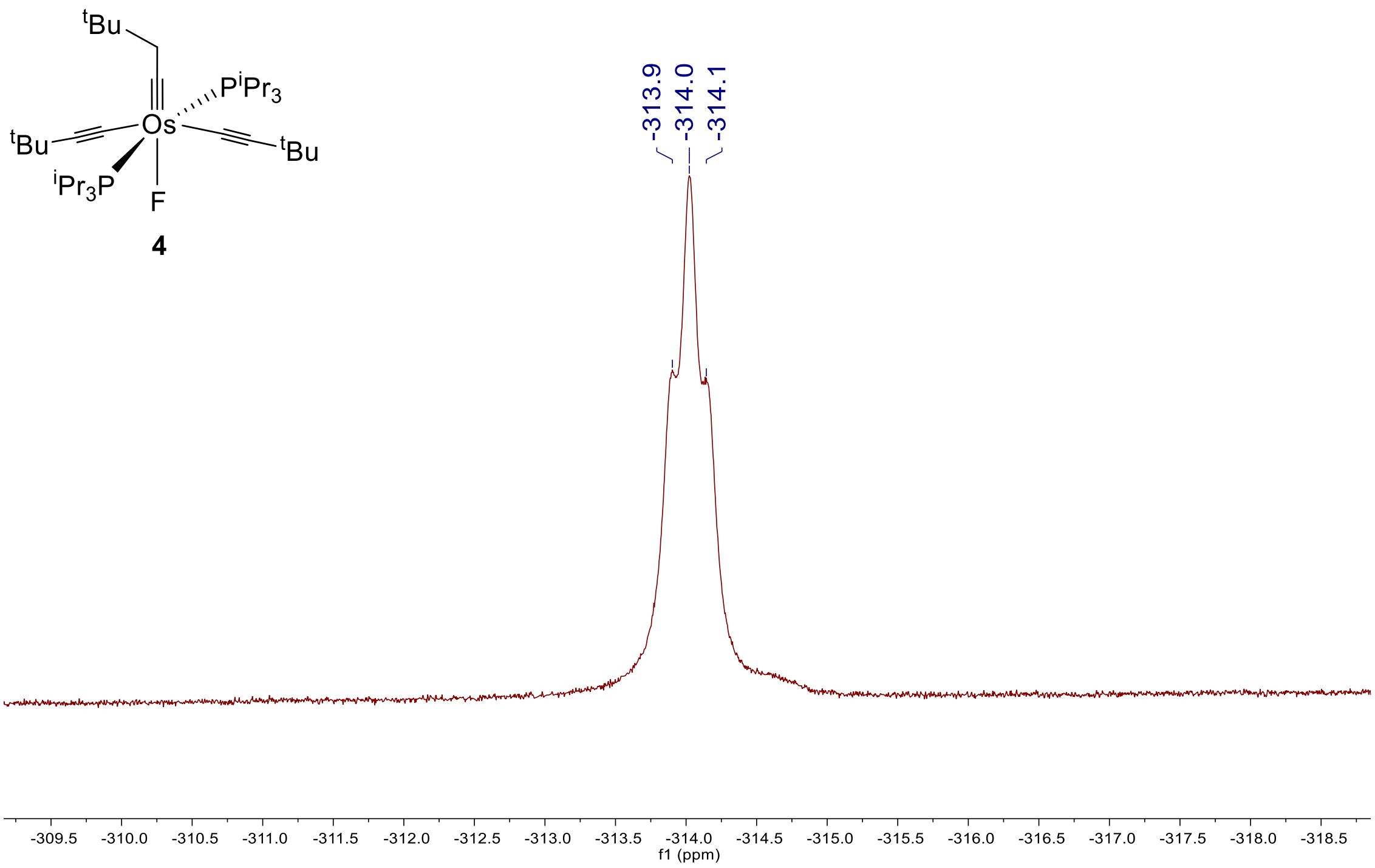

Figure S17. ^{19}F NMR spectrum (376.46 MHz, CD_2Cl_2, 298 K) of complex **4**.

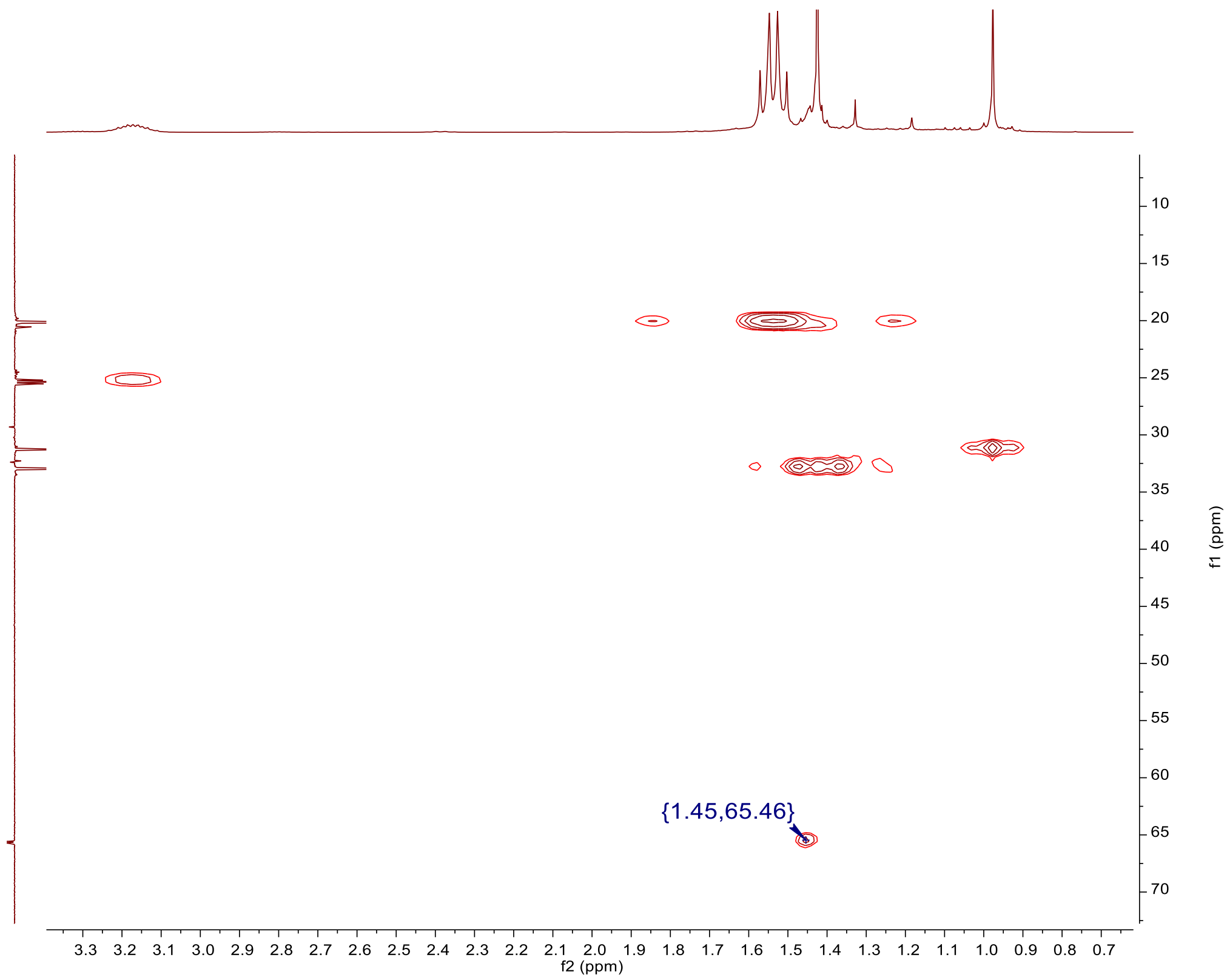

Figure S18. HSQC{^{1}H, $^{31}C(^{1}H)$} (C_6D_6, 298 K) of complex **4** displaying the cross-peak between the resonances corresponding to the CH_2 moiety of the carbyne ligand.

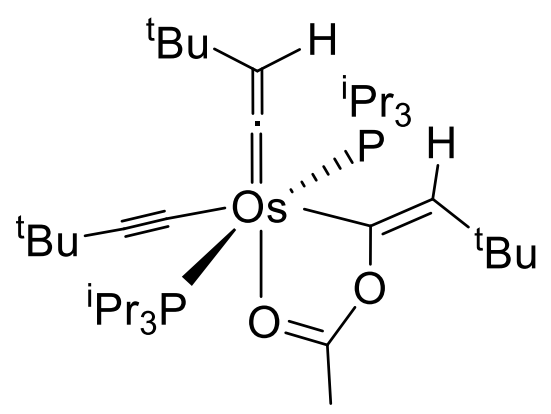

5

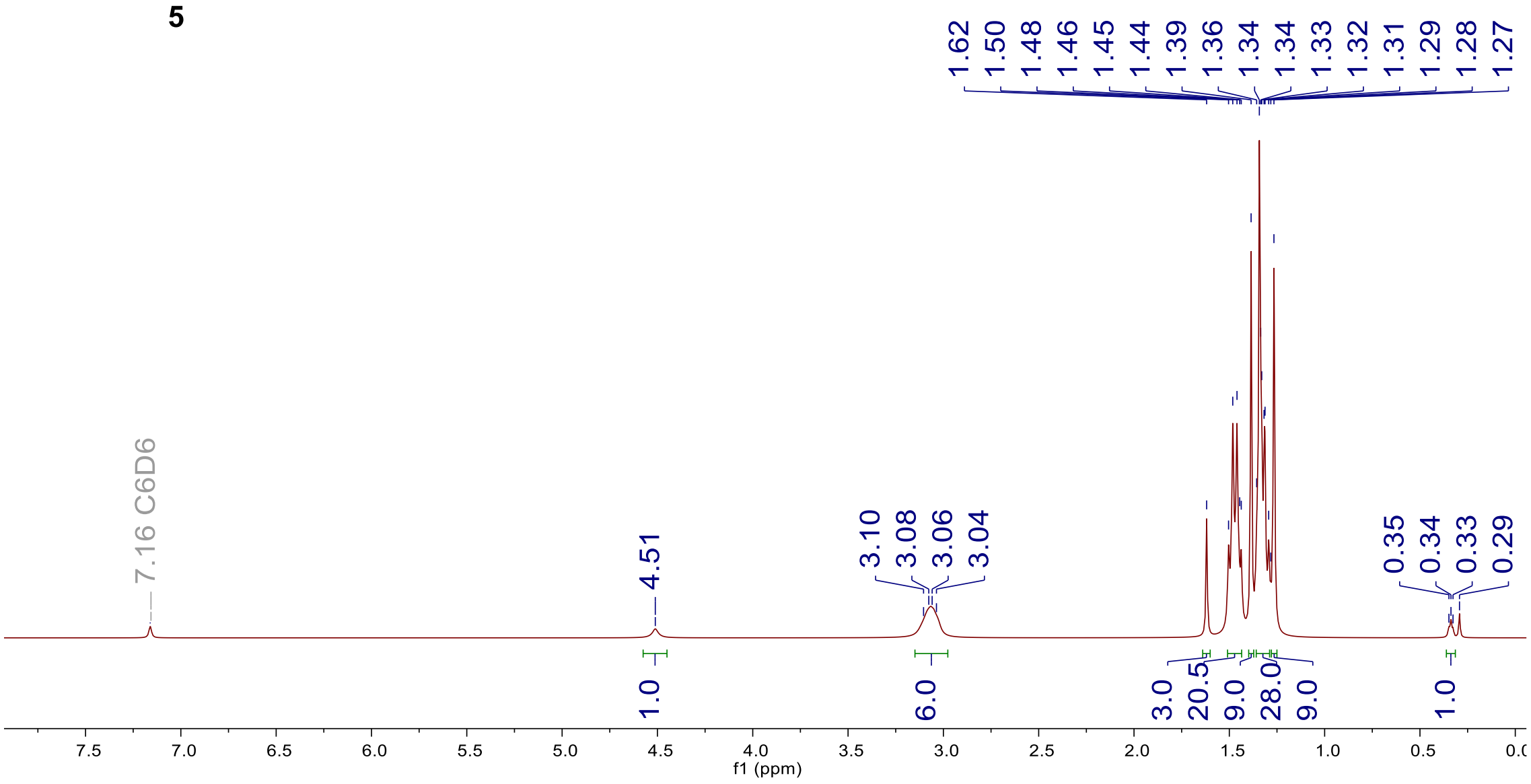

Figure S19. ^{1}H NMR spectrum (300.13 MHz, C_6D_6, 298 K) of complex **5**.

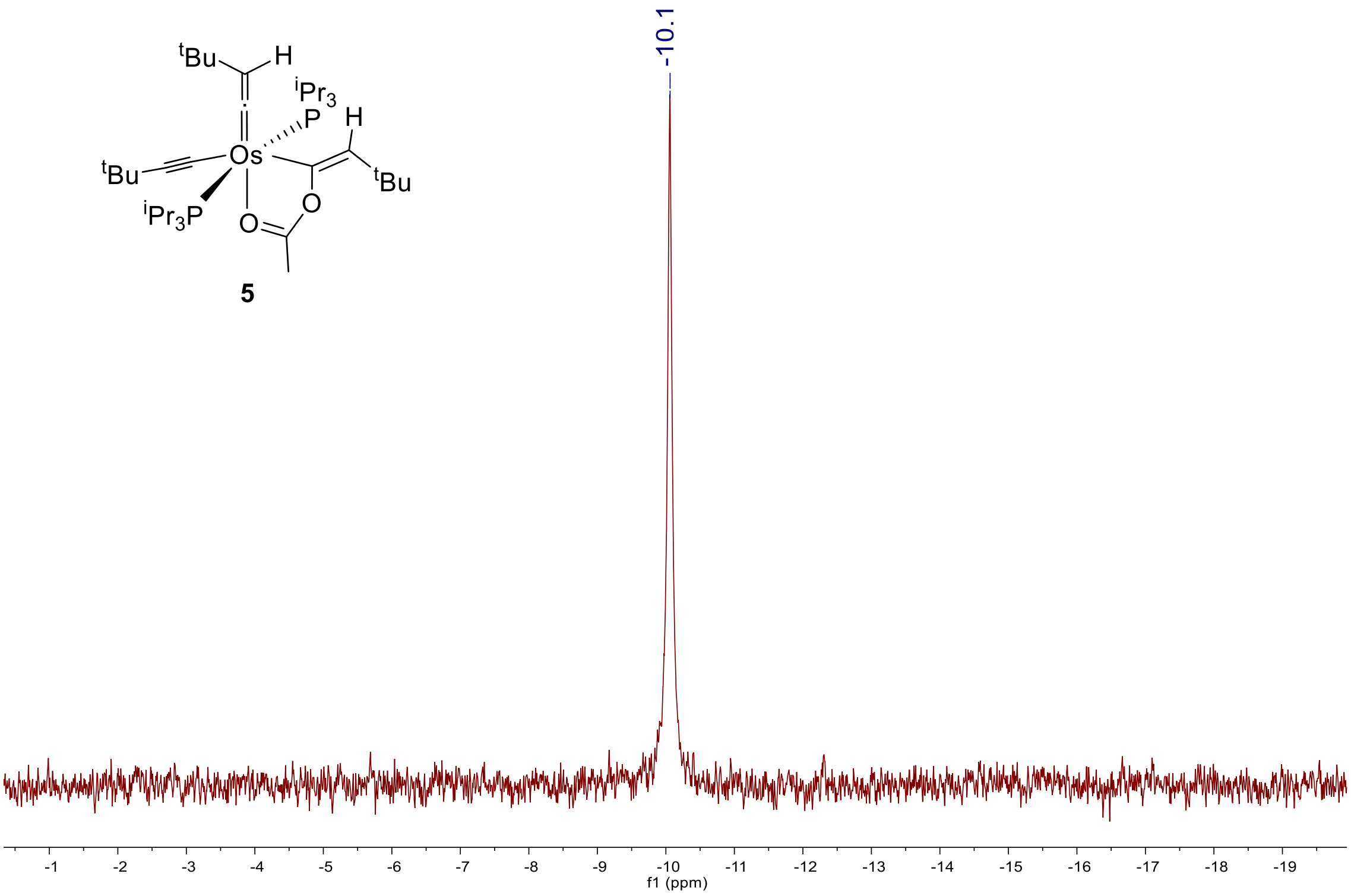

Figure S20. $^{31}P\{^{1}H\}$ NMR spectum (121.50 MHz, C_6D_6, 298 K) of complex **5**.

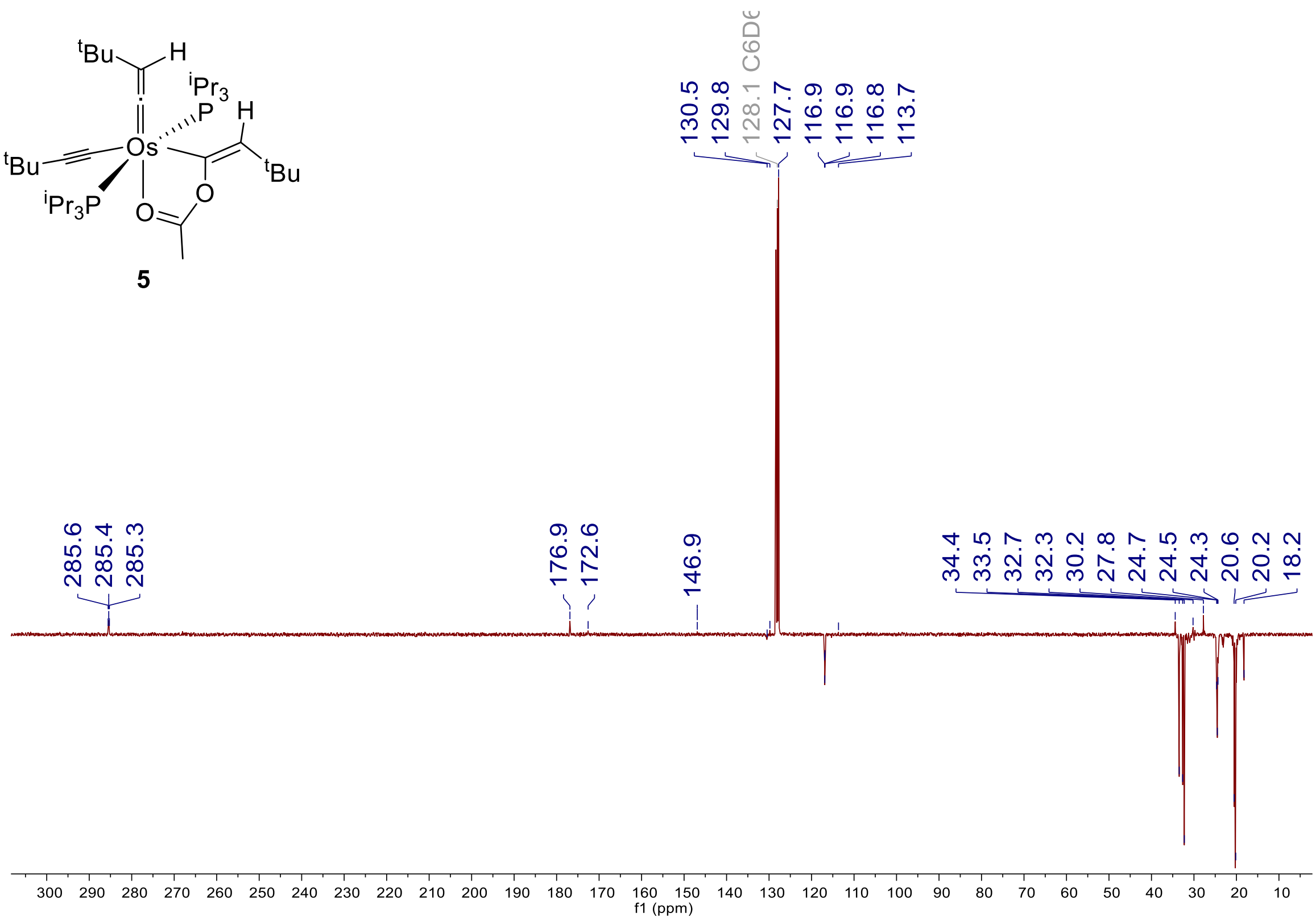

Figure S21. $^{13}C\{^{1}H\}$ APT NMR spectrum (75.48 MHz, C_6D_6, 298 K) of complex **5**.

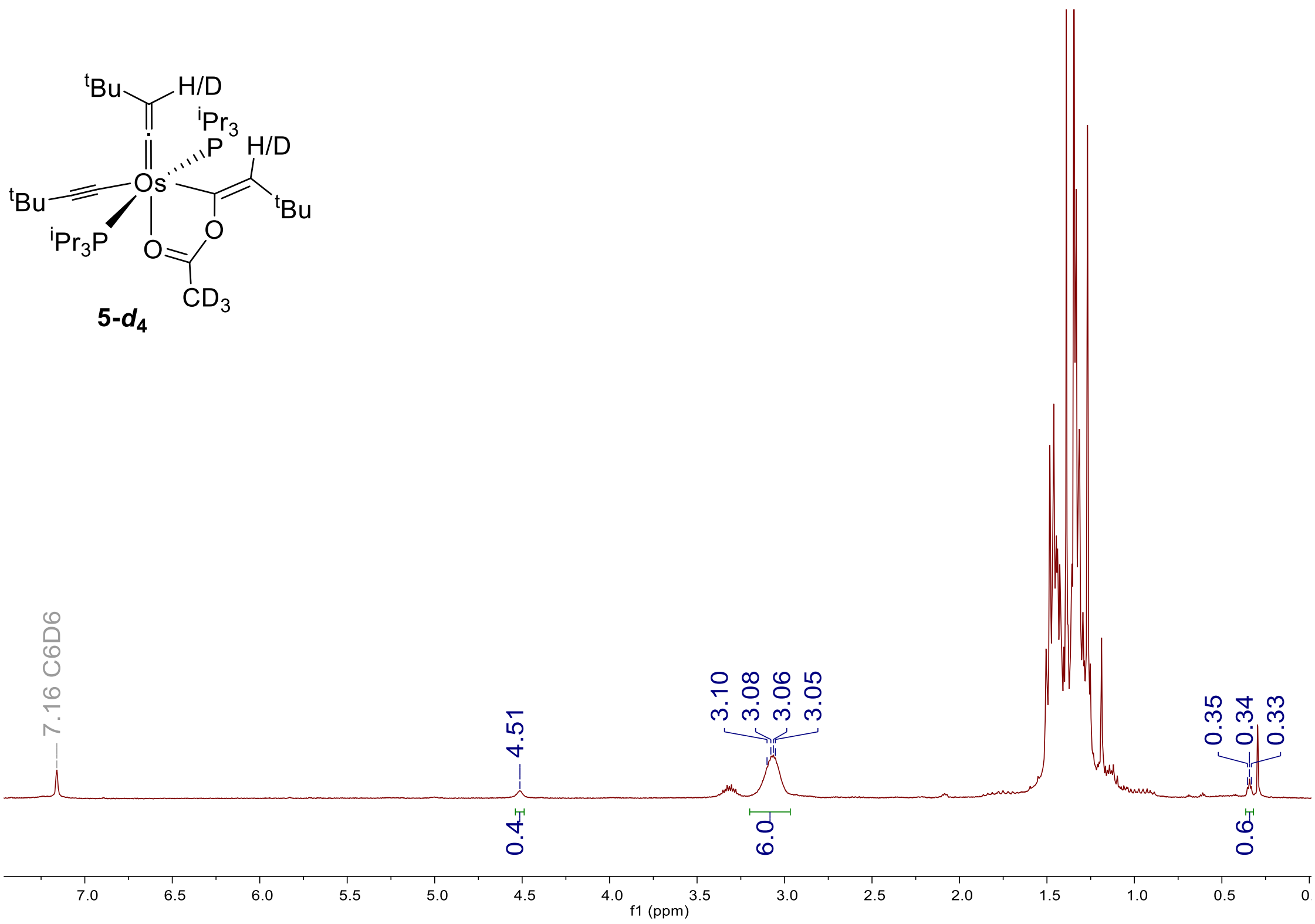

Figure S22. ^{1}H NMR spectrum (300.13 MHz, C_6D_6, 298 K) of complex **5-*d*4**.

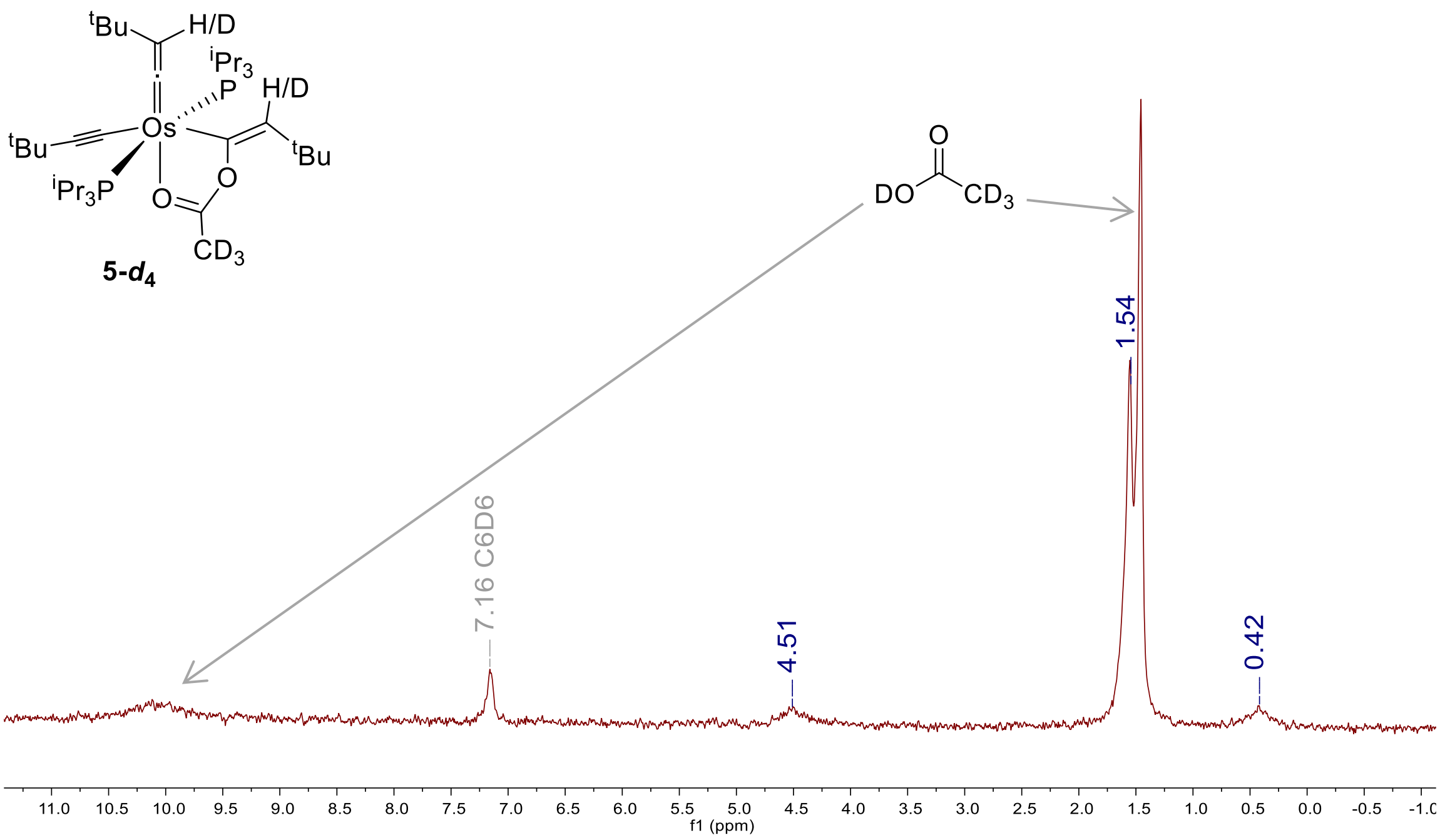

Figure S23. ^{3}H NMR spectrum (300.13 MHz, C_6D_6, 298 K) of complex **5-*d*4**.

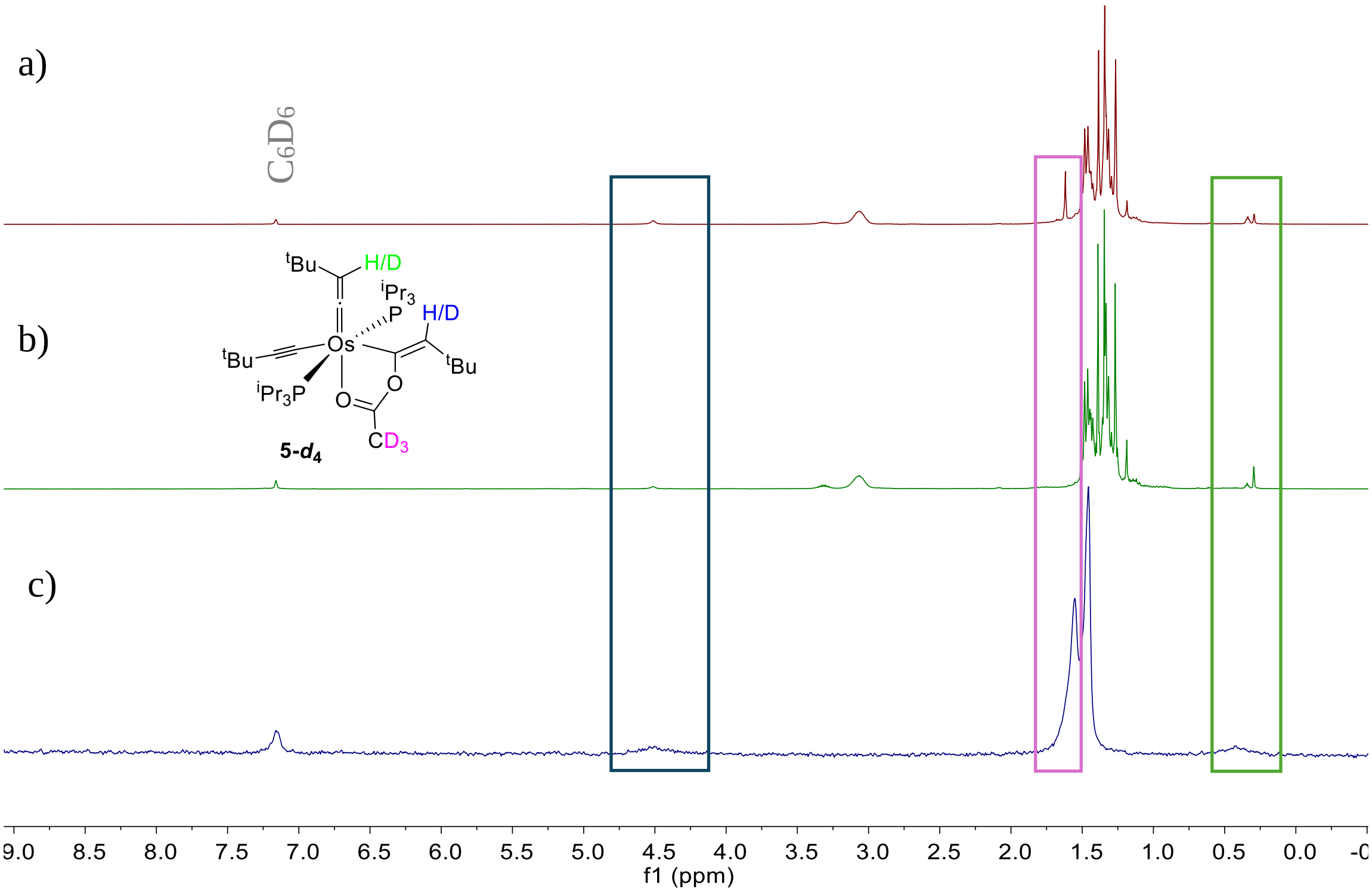

Figure S24. a) ^{1}H NMR spectrum (300.13 MHz, C_6D_6, 298 K) of complex **5**, b) ^{1}H NMR spectrum (300.13 MHz, C_6D_6, 298 K) of complex **5-*d*₄**, c) ^{2}H NMR spectrum (46.07 MHz, C_6H_6, 298 K) of complex **5-*d*₄**.

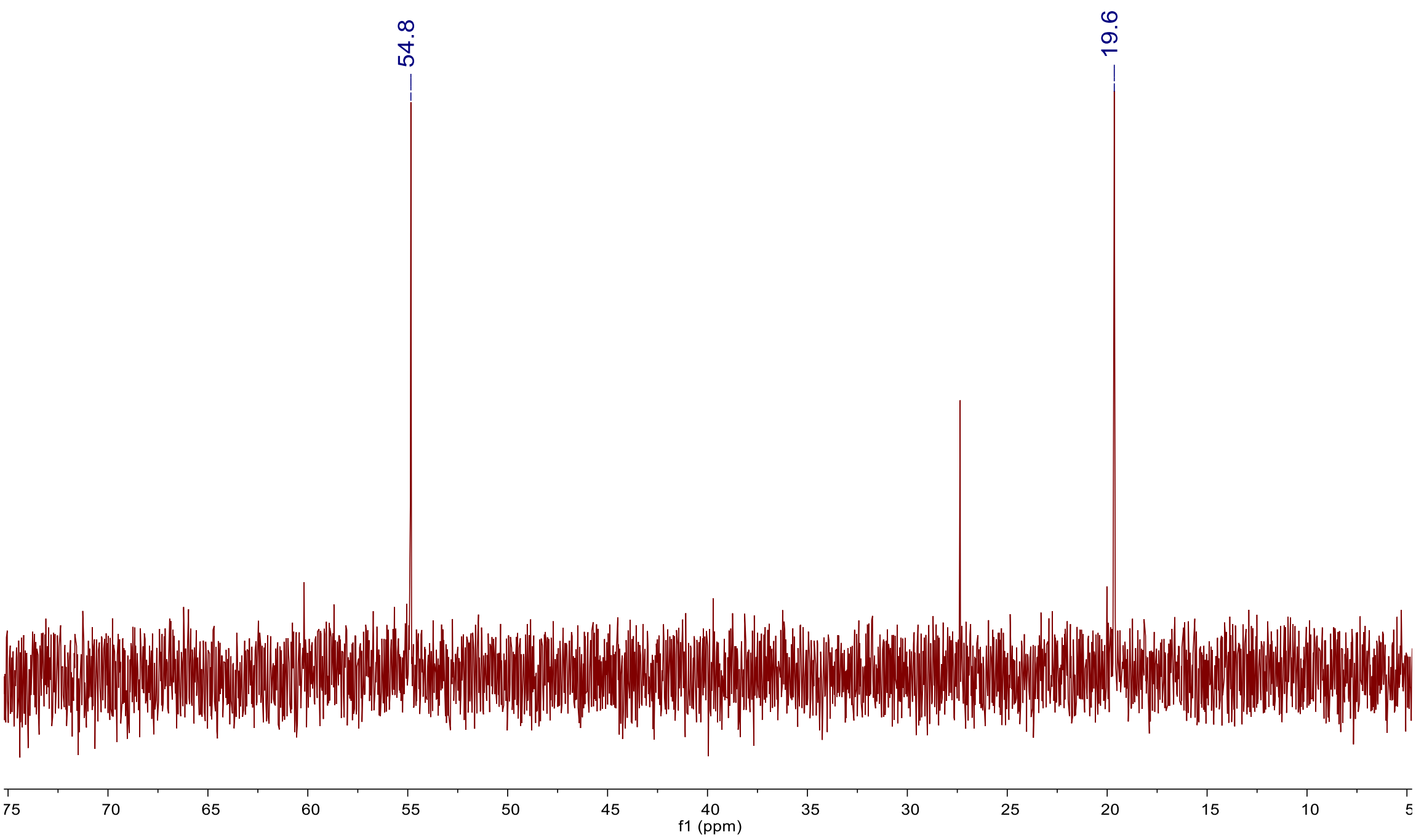

Figure S25. ^{31}P{^{1}H} NMR spectrum (121.50 MHz, C_6D_6, 298 K) of the crude of the reaction of 2-aminopyridine with complex **2**.

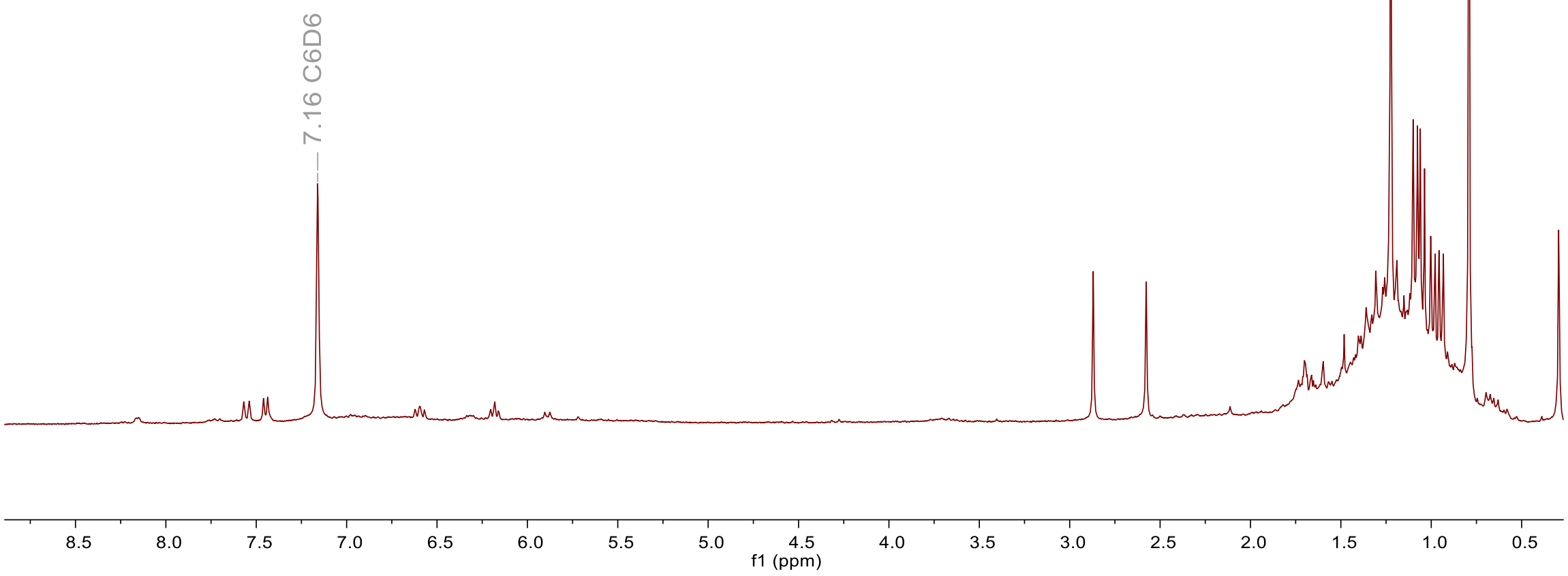

Figure S26. a) ^{1}H NMR spectrum (300.13 MHz, C_6D_6, 298 K) of the crude of the reaction of 2-aminopyridine with complex **2**.

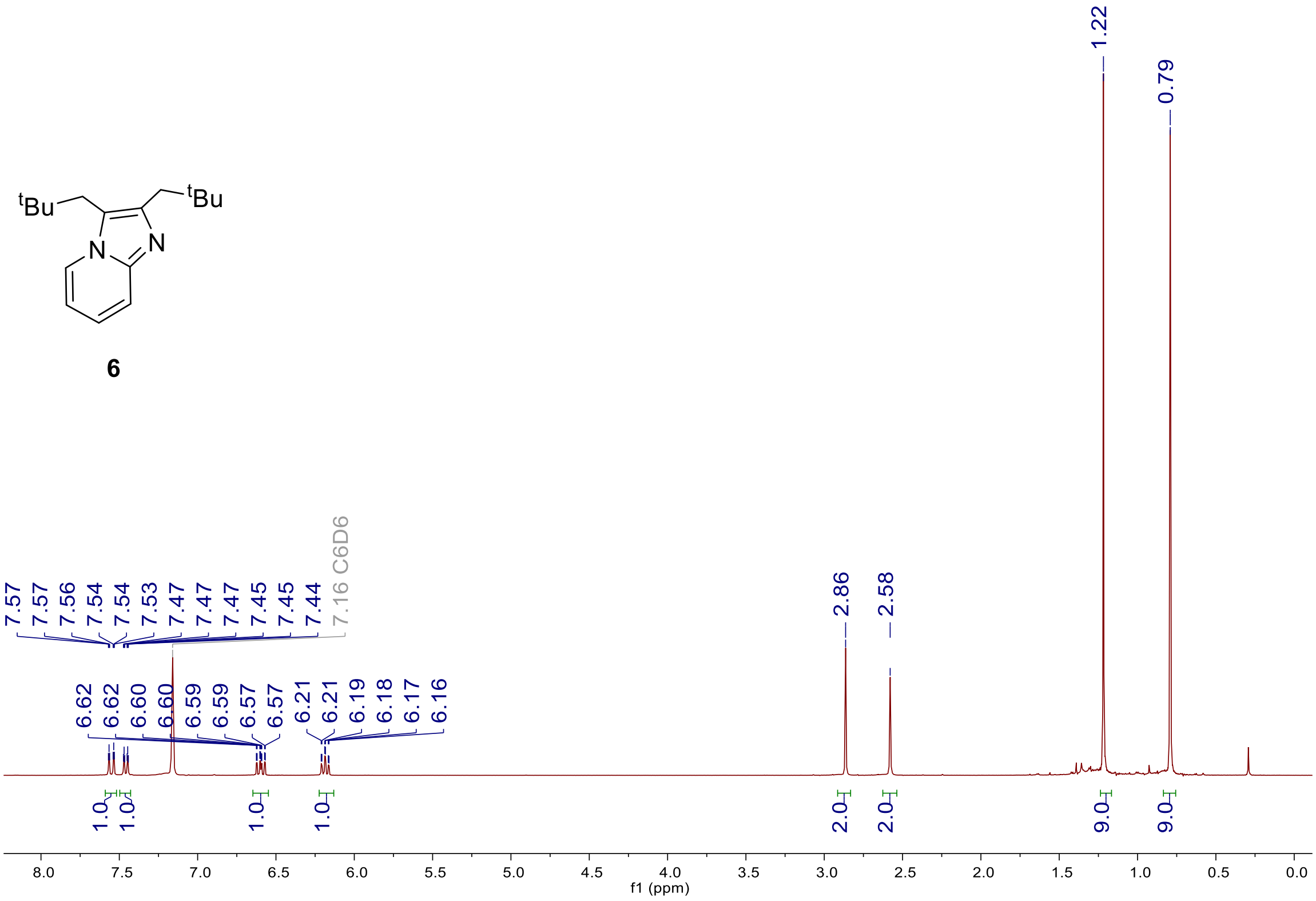

Figure S27. ^{1}H NMR spectrum (300.13 MHz, C_6D_6, 298 K) of 2,3-dineopentylimidazo[1,2-*a*]pyridine (**6**).

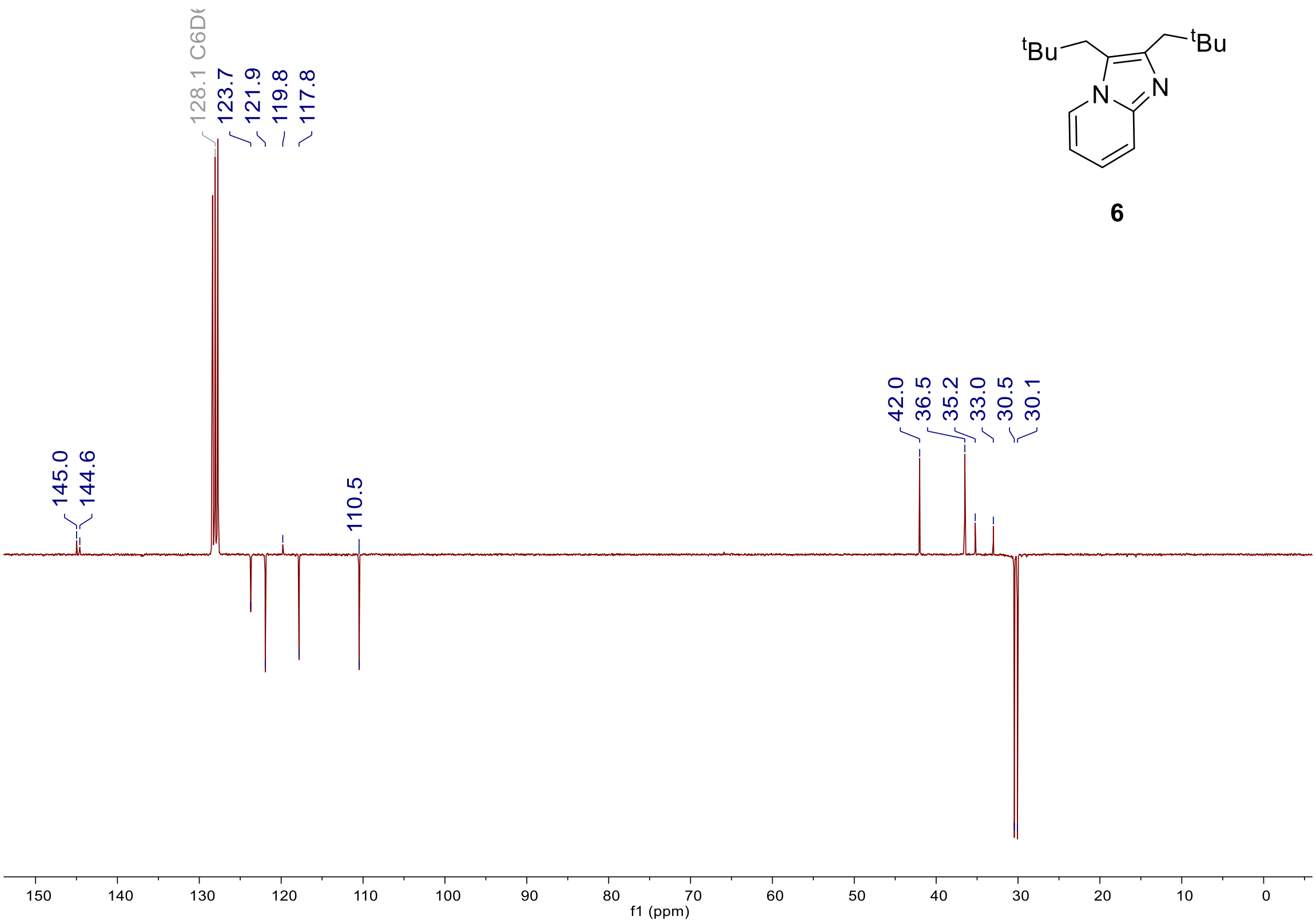

Figure S28. $^{13}C\{^1H\}$ APT NMR spectrum (75.48 MHz, C_6D_6, 298 K) of 2,3-dineopentylimidazo[1,2-*a*]pyridine (**6**).

• References

(1) Aracama, M.; Esteruelas, M. A.; Lahoz, F. J.; López, J. A.; Meyer, U.; Oro, L. A.; Werner, H. Synthesis, Reactivity, Molecular Structure, and Catalytic Activity of the Novel Dichlorodihydridoosmium(IV) Complexes $OsH_2C1_2(PR_3)_2$ (PR_3 = P-*i*-Pr_3, PMe-*t*-Bu_2). *Inorg. Chem.* **1991**, *30,* 288-293.

(2) Blessing, R. H. *Acta Crystallogr.* **1995**, *A51*, 33. SADABS: Area-detector absorption correction; Bruker- AXS, Madison, WI, 1996.

(3) SHELXL-2019/6. Sheldrick, G. M. *Acta Cryst.* **2008**, *A64*, 112-122.

(4) (a) Lee, C.; Yang, W.; Parr, R. G. Development of the Colle-Salvetti correlationenergy formula into a functional of the electron density. *Phys. Rev. B* **1988**, *37*, 785-789. (b) Becke, A. D. Density-functional exchange-energy approximation with correct asymptotic behavior. *J. Chem. Phys.* **1993**, *98*, 5648-5652. (c) Stephens, P. J.; Devlin, F. J.; Chabalowski, C. F.; Frisch, M. J. Ab Initio Calculation of Vibrational Absorption and Circular Dichroism Spectra Using Density Functional Force Fields. *J. Phys. Chem.* **1994**, *98*, 11623-11627.

(5) Grimme, S.; Antony, J.; Ehrlich, S.; Krieg, H. A consistent and accurate ab initio parametrization of density functional dispersion correction (DFT-D) for the 94 elements H-Pu. *J. Chem. Phys.* **2010**, *132*, 154104.

(6) Gaussian 09, Revision D.01, Frisch, M. J.; Trucks, G. W.; Schlegel H. B.; Scuseria, G. E.; Robb, M. A.; Cheeseman, J. R.; Scalmani, G.; Barone, V.; Mennucci, B.; Petersson, G. A.; Nakatsuji, H.; Caricato, M.; Li, X.; Hratchian, H. P.; Izmaylov, A. F.; Bloino, J.; Zheng, G.; Sonnenberg, J. L.; Hada, M.; Ehara, M.; Toyota, K.; Fukuda, R.; Hasegawa, J.; Ishida, M.; Nakajima, T.; Honda, Y.; Kitao, O.; Nakai, H.; Vreven, T.; Montgomery, J. A.; Peralta, Jr., J. E.; Ogliaro, F.; Bearpark, M.; Heyd, J. J.; Brothers, E.; Kudin, K. N.; Staroverov, V. N.; Keith, T.; Kobayashi, R.; Normand, J.; Raghavachari, K.; Rendell, A.; Burant, J. C.; Iyengar, S. S.; Tomasi, J.; Cossi, M.; Rega, N.; S43 Millam, J. M.; Klene, M.; Knox, J. E.; Cross, J. B.; Bakken, V.; Adamo, C.; Jaramillo, J.; Gomperts, R.; Stratmann, R. E.; Yazyev, O.; Austin, A. J.; Cammi, R.; Pomelli, C.; Ochterski, J. W.; Martin, R. L.; Morokuma, K.; Zakrzewski, V. G.; Voth, G. A.; Salvador, P.; Dannenberg,

J. J.; Dapprich, S.; Daniels, A. D.; Farkas, O.; Foresman, J. B.; Ortiz, J. V.; Cioslowski, J.; Fox, D. J. Gaussian, Inc., Wallingford CT, 2013.

(7) Andrea, D.; Häußermann, U. M.; Dolg, M.; Stoll, H.; Preuss, H. Energy adjusted ab initio pseudopotentials for the second and third row transition elements. *Theor. Chim. Acta* **1990**, *77*, 123-141.

(8) Ehlers, A. W.; Bohme, M.; Dapprich, S.; Gobbi, A.; Hollwarth, A.; Jonas, V.; Kohler, K. F.; Stegmann, R.; Veldkamp, A.; Frenking, G. A set of f-polarization functions for pseudo-potential basis sets of the transition metals SC-Cu, Y-Ag and La-Au. *Chem. Phys. Lett.* **1993**, *208*, 111-114.

(9) (a) Hehre, W. J.; Ditchfield, R.; Pople, J. A. Self-Consistent Molecular Orbital Methods. XII. Further Extensions of Gaussian-Type Basis Sets for Use in Molecular Orbital Studies of Organic Molecules. *J. Chem. Phys.* **1972**, *56*, 2257-2261. (b) Francl, M. M.; Pietro, W. J.; Hehre, W. J.; Binkley, J. S.; Gordon, M. S.; DeFrees, D. J.; Pople, J. A. Self-consistent molecular orbital methods. XXIII. A polarization-type basis set for second-row elements. *J. Chem. Phys.* **1982**, *77*, 3654-3665.

(10) Glendening, E. D.; Badenhoop, J. K.; Reed, A. E.; Carpenter, J. A.; Bohmann, C. M.; Weinhold, F. Theoretical Chemistry Institute, University of Wisconsin, Madison, WI (2018).
